# p120-catenin prevents multinucleation through control of MKLP1-dependent RhoA activity during cytokinesis

**DOI:** 10.1038/ncomms13874

**Published:** 2016-12-22

**Authors:** Robert A.H. van de Ven, Jolien S. de Groot, Danielle Park, Robert van Domselaar, Danielle de Jong, Karoly Szuhai, Elsken van der Wall, Oscar M. Rueda, H. Raza Ali, Carlos Caldas, Paul J. van Diest, Martin W. Hetzer, Erik Sahai, Patrick W.B. Derksen

**Affiliations:** 1 Department of Pathology, University Medical Center Utrecht, Heidelberglaan 100, 3584 CX Utrecht, The Netherlands; 2 Tumour Cell Biology Laboratory, Cancer Research UK London Research Institute, 44 Lincoln’s Inn Fields, London WC2A 3LY, UK; 3 Department of Molecular Cell Biology, Leiden University Medical Center, Einthovenweg 20, 2300 RC Leiden, The Netherlands; 4 Department of Internal Medicine, University Medical Center Utrecht, Heidelberglaan 100, 3584 CX Utrecht, The Netherlands; 5 Cancer Research UK Cambridge Institute, University of Cambridge, Li Ka Shing Centre, Cambridge, UK; 6 Department of Oncology, University of Cambridge, Addenbrooke’s Hospital, Cambridge, UK; 7 Cambridge Experimental Cancer Medicine Centre and NIHR Cambridge Biomedical Research Centre, Cambridge, UK; 8 Molecular and Cell Biology Laboratory, Salk Institute for Biological Studies, La Jolla, California 92037, USA

## Abstract

Spatiotemporal activation of RhoA and actomyosin contraction underpins cellular adhesion and division. Loss of cell–cell adhesion and chromosomal instability are cardinal events that drive tumour progression. Here, we show that p120-catenin (p120) not only controls cell–cell adhesion, but also acts as a critical regulator of cytokinesis. We find that p120 regulates actomyosin contractility through concomitant binding to RhoA and the centralspindlin component MKLP1, independent of cadherin association. In anaphase, p120 is enriched at the cleavage furrow where it binds MKLP1 to spatially control RhoA GTPase cycling. Binding of p120 to MKLP1 during cytokinesis depends on the N-terminal coiled-coil domain of p120 isoform 1A. Importantly, clinical data show that loss of p120 expression is a common event in breast cancer that strongly correlates with multinucleation and adverse patient survival. In summary, our study identifies p120 loss as a driver event of chromosomal instability in cancer.

## Abstract (web summary)

The tumour suppressor p120-catenin (p120) controls cadherin-based adhesion. Here, the authors demonstrate that p120 regulates cytokinesis through binding to the centralspindlin component MKLP1 and controls RhoA activity. Loss of p120 in cancer induces multinucleation and chromosomal instability, independent of cell-cell adhesion.

Tumour cells display chromosomal alterations including loss and gain of complete chromosomes termed aneuploidy^1^,^2^. It is thought that chromosomal instability (CIN) facilitates tumour plasticity and adaptation to changing environments through Darwinian selection processes. A number of mechanisms contribute to CIN including defects in the DNA damage response, mitotic checkpoints, and cytokinesis. Formation of malignant cells from binucleated cells is caused by the inheritance of extra centrosomes, which induces chromosome segregation errors during subsequent cell divisions^1^,^3^. Additional mechanisms include non-genetic pathways like steric hindrance of furrow ingression by carcinogens, or cell-in-cell structures derived through a process termed entosis^4^. Moreover, genetic alterations or loss of tumour suppressors can induce cytokinesis defects and subsequently drive malignant transformation^5^,^6^. Cytokinesis is intricately balanced through spatiotemporal recruitment and activation of the proteins that form the actomyosin contractile ring and drive cleavage furrow ingression^7^. Centralspindlin, a tetrameric complex composed of MgcRacGAP and the motor protein MKLP1 (KIF23), localizes to the central spindle where it regulates balanced actomysosin contractility during cytokinesis^8^,^9^,^10^. Recruitment of the RhoGEF Ect2 to the centralspindlin complex drives local activation of the small GTPase RhoA, which controls actomyosin contractility and subsequent furrow ingression^11^,^12^,^13^. Rapid RhoA GTPase cycling depends on the presence of positive and negative regulators and limits lateral diffusion to maintain a restricted active Rho zone essential for proper cytokinesis^14^,^15^,^16^.

Loss of cell–cell adhesion is commonly observed in human cancer upon inactivation of the Adherens Junction (AJ) components E-cadherin or p120-catenin^17^,^18^,^19^,^20^,^21^,^22^. Previously, we demonstrated that mammary-specific inactivation of E-cadherin causes the development and progression of invasive lobular breast carcinoma (ILC)^23^,^24^. Cortical stabilization of E-cadherin depends on binding of p120-catenin to the intracellular juxtamembrane domain^25^,^26^. Although conditional ablation of p120 causes formation of metastatic breast carcinoma in mice, it does not lead to ILC but instead induces the formation of metastatic high-grade ductal-type carcinoma^22^. These findings demonstrated that loss of p120 expression has differential functional consequences during tumour initiation and progression that may occur independently of cell–cell adhesion.

Here we show that loss of p120 is causal to the formation of multinucleated and chromosomally unstable tumour cells in mouse and human cancer models. Our data reveal that p120 is enriched at the equatorial cortex during cytokinesis where it regulates the spatiotemporal activity of RhoA. In addition, we show that p120 modulates RhoA GTPase cycling through isoform-specific binding to MKLP1. Altogether, our data reveal an unprecedented function for p120 in the regulation of cytokinesis and provide evidence that loss of p120 supports tumour progression through loss of cell–cell adhesion and induction of CIN.

## Results

### Loss of p120 induces multinucleation and contributes to CIN

**Figure 1:**
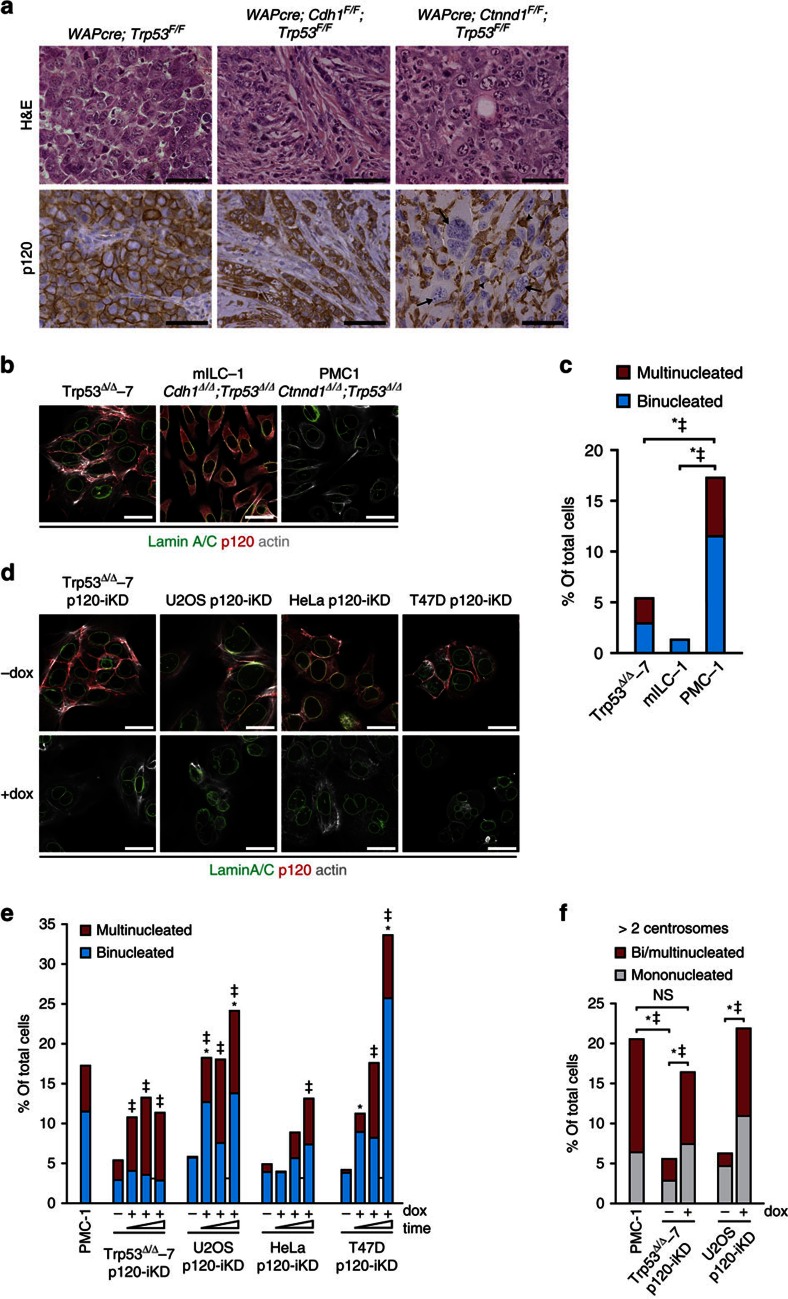
Loss of p120 induces multinucleation and chromosomal instability. (**a**) Mammary-specific conditional female mice mutant for p120 and p53 (*WAPcre;Ctnnd1*^*F/F*^*;Trp53*^*F/F*^), E-cadherin and p53 (*WAPcre;Cdh1*^*F/F*^*;Trp53*^*F/F*^) or p53 alone (*WAPcre;Trp53*^*F/F*^) were stained for p120. Note the overt multinucleation in the p120 knockout mammary carcinoma cells (arrow) and the prominent influx of p120 expressing immune cells (macrophages; arrow head). Tissue architecture and nuclear morphology were visualized by H&E stainings. Scale bar, 50 μm. (**b**) Immunofluorescence (IF) imaging of cell lines (Trp53^Δ/Δ^-7, mILC-1 and PMC-1) derived from the corresponding tumours shown in **a**. Scale bar, 10 μm. (**c**) Quantification of bi- and multinucleation in the cell lines shown in **b**. Statistical significance was determined using the *χ*^2^-test. *P<0.05/^‡^*P*<0.05 (binucleation and multinucleation, respectively). (**d**) Control (-dox) and dox-treated (+dox) mouse and human cancer cell lines transduced with an inducible p120KD were stained for p120 and Lamin A/C. Scale bar, 10 μm. (**e**) Quantification of bi- and multinucleation of the p120-iKD cell lines shown in **d** over three consecutive passages in the absence or presence of dox. At least 200 control and dox-treated cells were analysed every passage. (**f**) Quantification of centrosome numbers in interphase PMC-1, Trp53^Δ/Δ^-7 p120-iKD and U2OS p120-iKD cells. Centrosome numbers were determined by IF staining for γ-tubulin. Statistical significance in **c**,**e** and **f** was determined using the χ^2^-test. *P<0.05/^‡^*P*<0.05 (binucleation and multinucleation respectively). NS, not significant.

Previous work in our lab has shown that loss of the AJ through somatic inactivation of either E-cadherin or p120 in the context of p53 inactivation is causal to the development of metastatic mammary carcinomas^22^,^23^,^24^. However, while loss of E-cadherin and p53 in *WAPcre;Cdh1*^*F/F*^*;Trp53*^*F/F*^ female mice induced the formation of mouse ILC (mILC), homozygous dual inactivation of p120 and p53 (*WAPcre;Ctnnd1*^*F/F*^*;Trp53*^*F/F*^) induced the formation of high-grade invasive ductal-type carcinoma (Fig. 1a). Interestingly, we observed a high incidence of multinucleated tumour cells and overall severe nuclear atypia in p120-deficient mammary carcinomas, whereas E-cadherin and p53-deficient tumours did not show overt nuclear abnormalities (Fig. 1a). To further examine this we made use of mammary carcinoma cell lines derived from our mouse models and observed a high frequency of bi- and multinucleation in PMC-1 cells (*Ctnnd1*^*Δ/Δ*^*;Trp53*^*Δ/Δ*^) compared with mILC-1 (*Cdh1*^*Δ/Δ*^*;Trp53*^*Δ/Δ*^) and Trp53^Δ/Δ^-7 cells (Fig. 1b,c). We next visualized chromosomal content using multicolour combined binary ratio (COBRA)-FISH and observed that virtually all PMC-1 cells showed a numerical increase of chromosomal content (ranging from 4N to 8N), whereas most p120-proficient Trp53^Δ/Δ^-7 and mILC-1 cell lines displayed a near diploid content (Supplementary Fig. 1a; Supplementary Table 1). To establish causality we used a doxycyclin (dox)-inducible shRNA expression vector to silence p120 (p120-iKD; Supplementary Fig. 1b) and observed a robust increase in bi- and multinucleation in cell lines of different origin (Fig. 1d,e). The frequency of bi- and multinucleation in dox-treated p120-iKD cells accumulated up to three passages suggesting a continuous process and/or a selective advantage for the multinucleated state (Fig. 1e). Multinucleated cells are chromosomally unstable due to the inheritance of extra centrosomes that can cause chromosome segregation defects during subsequent cell divisions^3^. Indeed, we observed a significant increase in centrosome numbers in PMC-1 and dox-treated p120-iKD cell lines and detected an increase of mononucleated cells expressing more than two centrosomes (Fig. 1f), indicating that these cells were derived from bi- or multinucleated cells but reverted to a mononuclear state. Altogether, our results show that loss of p120 is causal to multinucleation and contributes to CIN in cancer cells.

### p120 loss induces multinucleation independent of cadherins

**Figure 2:**
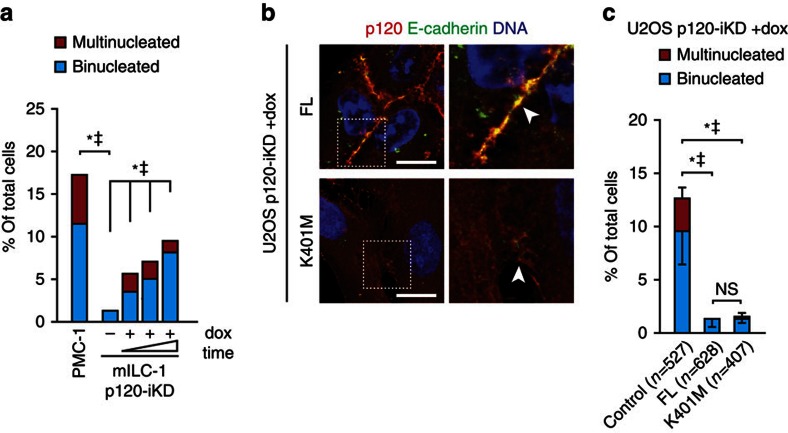
Multinucleation induced by p120 loss occurs independent of cadherin association. (**a**) Quantification of bi- and multinucleation in control and dox-treated E-cadherin-deficient mILC-1 p120-iKD cells over three consecutive passages of dox-treated in the presence of dox. Statistical significance was determined using the χ^2^-test. *P<0.05/^‡^*P*<0.05 (binucleation and multinucleation respectively). (**b**) IF of p120 and E-cadherin in dox-treated U2OS p120-iKD cells reconstituted with FL p120-1A or p120-1A K401M. Right panels show magnifications of representative areas denoted by the dotted squares. Scale bar, 10 μm. (**c**) Quantification of bi- and multinucleation in dox-treated U2OS p120-iKD reconstituted with empty vector (control), p120-1A FL or p120-1A K401M. Statistical significance was determined using the Student’s *t*-test. Shown are data from three independent experiments. Results are expressed as mean±s.d. *P<0.05/^‡^*P*<0.05 (binucleation and multinucleation, respectively).

Because multinucleation was increased in p120-null mammary carcinomas compared with E-cadherin-deficient tumours, it suggested that the induction of multinucleation upon p120 loss occurs independently of its role at the AJ and/or p53 inactivation. To substantiate the AJ-independent option we depleted p120 in mILC-1 cells, which are deficient for classical cadherins (Supplementary Fig. 1b) and observed an accumulation of bi- and multinucleated cells (Fig. 2a). Moreover, we reconstituted dox-treated U2OS p120-iKD cells with either full-length (FL) p120-1A or the p120-1A K410M mutant that contains a substitution at lysine 401 that disrupts binding to classical cadherins^27^ (Fig. 2b; Supplementary Fig. 1c). In line with our hypothesis, we observed that p120-1A K401M fully rescued the formation of bi- and multinucleated cells upon p120 loss (Fig. 2c), confirming that loss of p120 induces multinucleation through a process that is independent of cadherin association.

### Loss of p120 induces cytokinesis defects

**Figure 3:**
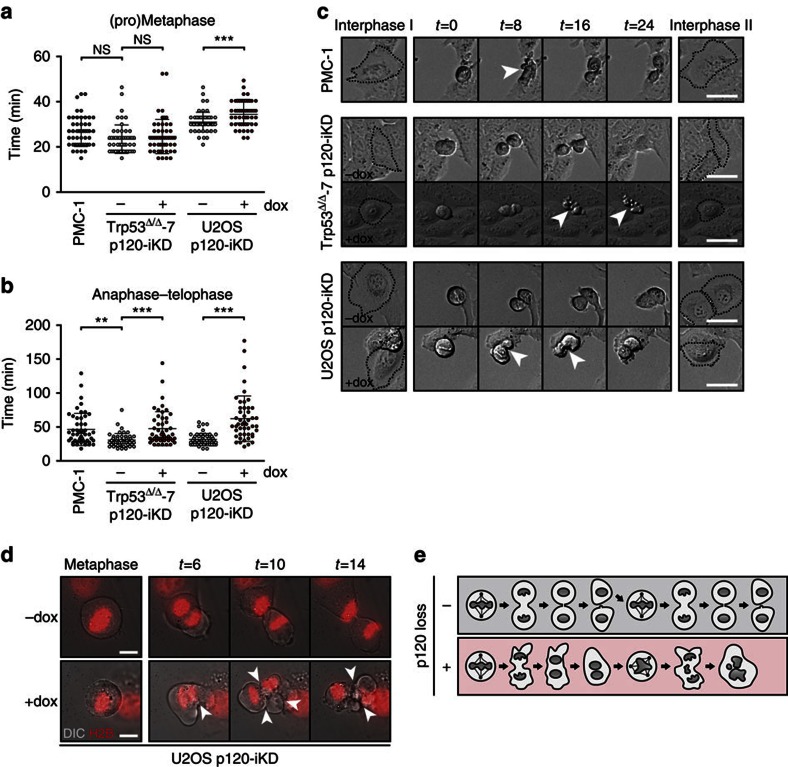
Loss of p120 induces cytokinesis defects. (**a**,**b**) Quantifications of the length of (**a**) early mitosis (pro-metaphase and metaphase) and (**b**) late mitosis/cytokinesis (anaphase and telophase) in PMC-1, control and dox-treated Trp53^Δ/Δ^-7 p120-iKD and U2OS p120-iKD cell lines. Statistical significance was determined using the Student’s *t*-test. *P<0.05, **P<0.01, ***P<0.001. (**c**) Time-lapse imaging of PMC-1, and control and dox-treated Trp53^Δ/Δ^-7 p120-iKD and U2OS p120-iKD cells. Shown are 10X DIC images. Scale bar, 25 μm. (**d**) High-magnification time-lapse imaging of H2B-mCherry expressing control and dox-treated U2OS p120-iKD. Shown are × 100 images. Arrowheads depict severe membrane deformation in dox-treated anaphase cells leading to cytokinesis failure. Scale bar, 10 μm. (**e**) Loss of p120 induces binucleation through cytokinesis defects during anaphase. During subsequent cell divisions, this can give rise to chromosomal instability and aneuploidy in cancer cells due to the presence of extra centrosomes.

Continuous defects during cytokinesis and/or abscission may represent the mechanism underlying the induction of multinucleation in p120-deficient cells. To investigate this we quantified the timing of mitosis in PMC-1 cells and p120-iKD cell lines. We did not detect defects during early mitosis (pro-metaphase and metaphase) in PMC-1 and Trp53^Δ/Δ^-7 p120-iKD, although the length of the early stages of mitosis in U2OS p120-iKD was slightly increased (Fig. 3a). However, the duration of late mitosis and cytokinesis (anaphase and telophase) was significantly increased in PMC-1 and both p120-iKD cell lines (Fig. 3b). In addition, we quantified the frequency of lagging chromosomes in U2OS p120-iKD cells and did not observe an increase after one passage on dox (−dox: 10/34; +dox: 6/23; *P*=0.428) indicating that the induction of CIN through loss of p120 is not caused by early chromosome segregation errors. However, we observed severe membrane deformations specifically during anaphase in PMC-1 and p120-iKD cells, ultimately resulting in cytokinesis failure and the formation of a binucleated cell (Fig. 3c). High-magnification time-lapse imaging showed rapid and prolonged membrane oscillations starting at the onset of anaphase that suggested aberrations in actomyosin contraction (Fig. 3d; Supplementary Movies 1 and 2). Together, our data show that loss of p120 induces continuous cytokinesis defects characterized by membrane oscillations that ultimately lead to the generation of chromosomally unstable multinucleated cancer cells (Fig. 3e).

### p120 controls RhoA activity during cytokinesis

**Figure 4:**
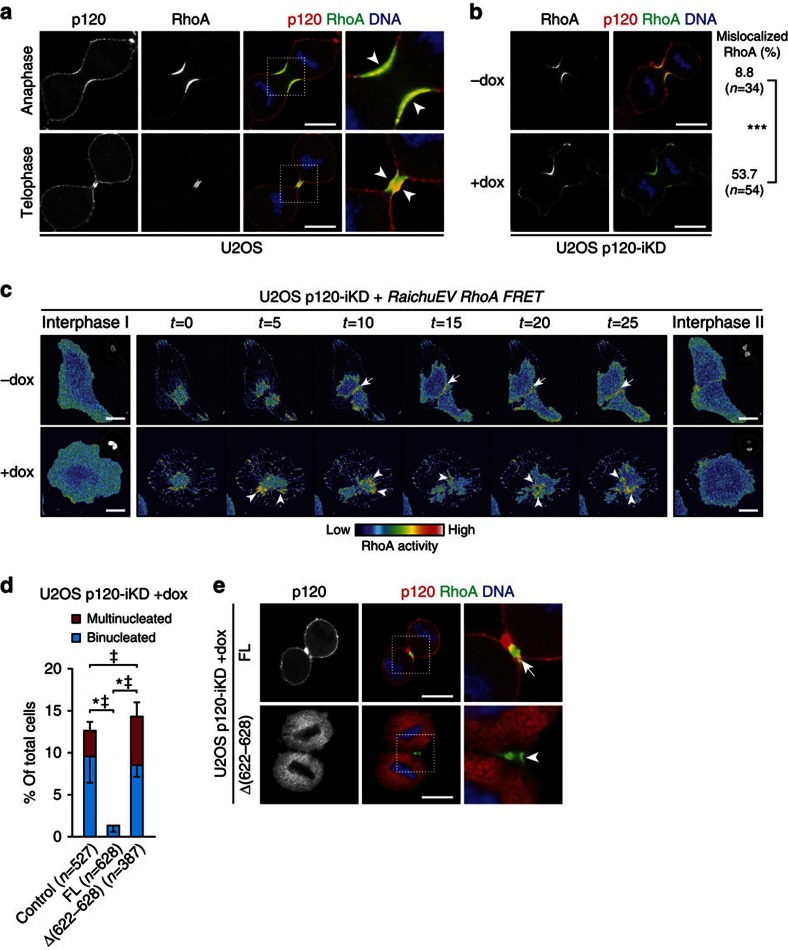
p120 regulates RhoA activity on the equatorial cortex during cytokinesis. (**a**) IF for p120 and RhoA in anaphase and telophase U2OS cells. Note the accumulation of p120 and co-localization on the equatorial cortex (arrowheads). Right panels show magnifications of representative areas denoted by the dotted squares. Scale bar, 10 μm. (**b**) IF for p120 and RhoA in control and dox-treated U2OS p120-iKD cells. Scale bar, 10 μm. Quantifications show the percentage of control and dox-treated U2OS p120-iKD cells with RhoA-positive membrane protrusions during anaphase. ***P<0.001. Statistical significance was determined using the *χ*^2^-test. (**c**) Time-lapse stills of control and dox-treated U2OS p120-iKD cells expressing the RaichuEV RhoA FRET probe. Note the focused RhoA zone in control cells (upper panels; arrows), whereas RhoA activity is non-focused and oscillates in dox-treated U2OS p120-iKD cells leading to cytokinesis failure (lower panels; arrowheads). Insets show H2B-mCherry channels. Scale bar, 20 μm. (**d**) Quantification of bi- and multinucleation in dox-treated U2OS p120-iKD reconstituted with empty vector (control), FL p120-1A or the RhoA-binding mutant p120-1AΔ[622–628]. Statistical significance was determined using the Student’s *t*-test. Shown are the data from three independent experiments. Results are expressed as mean±s.d. *P<0.05/^‡^*P*<0.05 (binucleation and multinucleation, respectively). (**e**) IF for p120 and RhoA in dox-treated U2OS p120-IKD cells reconstituted with FL p120-1A or p120-1AΔ[622–628]. Note the absence of co-localization of RhoA and p120 in p120-1AΔ[622–628]-expressing cells (arrowhead) compared with cells reconstituted with p120-A FL (arrow). Scale bar, 10 μm.

Formation of the contractile ring and subsequent cleavage furrow ingression during cytokinesis depends on local accumulation and rapid GTPase cycling of RhoA at the equatorial cortex^14^,^15^,^28^. Interestingly, while p120 was cortically localized during anaphase and telophase, we observed distinct enrichment of p120 at the equatorial cortex and the midbody that overlapped with accumulation of RhoA (Fig. 4a; Supplementary Fig. 2a/b). On the basis of this and the finding that p120 has been suggested to inhibit Rho GTP loading^29^, we hypothesized that loss of p120 could result in aberrant RhoA activity during furrow ingression, analogous to the phenotype observed upon loss of RhoGAP function during cytokinesis^15^,^16^. In contrast to the highly focused RhoA localization at the equatorial cortex during anaphase in control U2OS p120-iKD cells, we observed that RhoA expression was aberrantly localized to distinct membrane protrusions in dox-treated U2OS p120-iKD cells (Fig. 4b). To assess RhoA activity during cytokinesis, we used the Rho-specific RaichuEV FRET probe^30^. In contrast to control U2OS p120-iKD cells, we detected oscillations of active RhoA during cytokinesis (*t*=5 and *t*=10) in dox-treated U2OS p120-iKD cells resulting in non-focused RhoA activity (*t*=20 and *t*=25), which induced cytokinesis failure and binucleation (Fig. 4c; Supplementary Movies 3 and 4). In addition, we observed similar aberrations in RhoA activity in PMC-1 cells and Trp53^Δ/Δ^-7 p120-iKD cells (Supplementary Fig. 3a,b). In agreement with the mislocalized activity of RhoA, we detected aberrant localization of phosphorylated MLC2 during cytokinesis in p120-deficient cells (Supplementary Fig. 3c) accompanied by rapid oscillations of F-actin accumulation at sites other than the cleavage furrow (Supplementary Fig. 3d; Supplementary Movies 5 and 6).

A small lysine-rich region in the Armadillo (ARM) domains of p120 (residues 622–628) controls the inhibition of RhoA activity^29^. To test whether p120 regulates cytokinesis through modulation of RhoA activity, we reconstituted dox-treated U2OS p120-iKD with a p120 mutant lacking this region (p120-1AΔ[622–628]). While removal of the lysine-rich region did not induce blebbing to the extent of p120 depletion, we observed that reconstitution with p120-1AΔ[622–628] did not rescue multinucleation, which shows that this p120 region is crucial for control of RhoA activity at the equatorial cortex during cytokinesis (Fig. 4d). While p120-1A FL and p120-1A K401M co-localized with RhoA at the equatorial cortex (Fig. 4e; Supplementary Fig. 3e), p120-1AΔ[622–628] did not position to the equatorial cortex but instead was exclusively localized to the cytosol (Fig. 4e). Altogether, these data show that proper cytokinesis depends on local modulation of RhoA-dependent actomyosin contraction by p120 at the equatorial cortex. As a result, p120 loss leads to non-focused RhoA activity during anaphase, which is causal to cytokinesis failure and multinucleation.

### Binding of p120 to MKLP1 controls balanced RhoA activity

**Figure 5:**
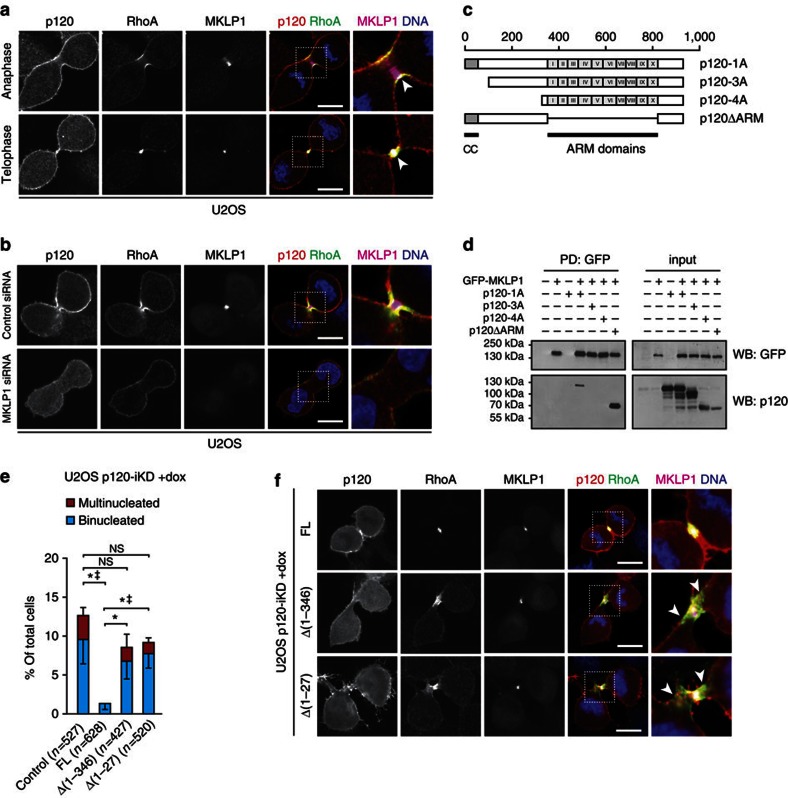
p120 interacts with MKLP1 to regulate focused RhoA activity during cytokinesis. (**a**) IF for p120, RhoA and MKLP1 in anaphase and telophase U2OS. Note the co-localization of p120, RhoA and MKLP1 at the equatorial cortex (arrowheads). Right panels show representative magnifications of representative areas denoted by the dotted squares. Scale bar, 10 μm. (**b**) IF for p120, RhoA and MKLP1 in U2OS cells transfected with control and MKLP1-specific siRNAs. Right panels show magnifications of representative areas denoted by the dotted squares. Scale bar, 10 μm. (**c**) Overview of the different p120 constructs used. CC=coiled-coil domains, ARM=Armadillo. Scale bar denotes the amino acid position. (**d**) Western blot showing the co-immunoprecipitations for GFP-MKLP1 and p120 constructs described in **c**. (**e**) Quantification of bi- and multinucleation in dox-treated U2OS p120-iKD reconstituted with empty vector (control), FL p120-1A, p120-1AΔ[1–346], and p120-1AΔ[1–27]. Statistical significance was determined using the Student’s *t*-test. Shown are the data from three independent experiments. Results are expressed as mean±s.d. *P<0.05/^‡^*P*<0.05 (binucleation and multinucleation, respectively). (**f**) IF for p120, RhoA and MKLP1 in dox-treated U2OS p120-iKD cells reconstituted with FL p120-1A, p120-1AΔ[1–346] and p120-1AΔ[1–27]. Note the broadened RhoA zone in cells reconstituted with p120-1AΔ[1–346] and p120-1AΔ[1–27] (arrowheads). Scale bar, 10 μm.

To unravel the mechanism that controls p120-dependent spatiotemporal regulation of RhoA activity during cytokinesis, we initially performed p120 immunoprecipitation and subsequent mass spectrometry on mILC-1 lysates to enrich for cadherin-independent binding partners (Supplementary Fig. 4a). Interestingly, we identified several established cytokinesis-related molecules (Supplementary Dataset 1). Of these, the strongest interaction was found between p120 and the centralspindlin component mitotic kinesin-like protein 1 (MKLP1; mascot score: 697). Centralspindlin is a tetrameric complex composed of MgcRacGAP and MKLP1 that accumulates at the spindle midzone during anaphase and functions as an essential regulator of cytokinesis through recruitment of the RhoGEF Ect2 (refs 11, 12, 31). Immunofluorescence microscopy confirmed co-localization of p120, RhoA and MKLP1 at the equatorial cortex during anaphase and telophase (Fig. 5a). To study the functional relevance of the interaction between p120, RhoA and MKLP1, we depleted MKLP1 by RNAi and induced cell cycle arrest to avoid early cytokinesis defects. Upon release we observed an impairment of cleavage furrow formation and ingression as described before^11^ (Fig. 5b). Depletion of MKLP1 abolished accumulation of RhoA specifically at the equatorial cortex leading to a broad primitive cleavage furrow (Fig. 5b). In addition, MKLP1-depleted cells showed an overall decrease in p120 expression at the equatorial cortex, suggesting that localization of p120 to this region depends on the presence of RhoA. Supporting this is our finding that our mass spectrometry data revealed RhoA as a low-hit p120 interactor (Supplementary Data 1), a finding we have confirmed by co-immunoprecipitation of endogenous p120 and RhoA using western blotting (Supplementary Fig. 4b). We therefore hypothesized that, although p120 is a global inhibitor of RhoA, the association between p120 and MKLP1 could provide the spatial cue that controls RhoA activity at the equatorial cortex during cytokinesis. To test this, we mapped the MKLP1-binding region in p120 by co-expressing GFP-MKLP1 with the naturally occurring p120 isoforms 1A, 3A, 4A, or a truncation mutant lacking all ARM domains (p120ΔARM) (Fig. 5c). Interestingly, only p120-1A and p120ΔARM precipitated with GFP-MKLP1, indicating that binding occurs through the N-terminal domain of p120, which includes the coiled-coil (CC) domain (Fig. 5d). To determine the significance of this interaction, we reconstituted dox-treated U2OS p120-iKD with either FL p120-1A, or N-terminal truncation mutants lacking the complete regulatory domain (p120-1AΔ[1–346]), or a CC truncation mutant (p120-1AΔ[1–27]; Supplementary Fig. 1c). Although the extent of aberrant actomyosin contraction was not as severe as in the p120 depleted cells, we observed that reconstitution with either p120-1AΔ[1–346] or p120-1AΔ[1–27] was not sufficient to rescue the bi- and multinucleation (Fig. 5e). Moreover, we observed that cells reconstituted with these truncation mutants displayed a broadened RhoA gradient during cytokinesis that was characterized by a lack of co-localization with p120 at the distal sides of the RhoA zone (Fig. 5f). Classical Rho GDI molecules act as inhibitors that mostly interact with Rho GTPases to prevent nucleotide exchange. Further, they can relocate Rho proteins from the membranes to the cytosol to prevent activation at membranes (reviewed in ref. 32). Although it is clear that p120 can control RhoA activity^29^,^33^,^34^,^35^, it is not a classical RhoGDI. Alternatively, p120 could therefore also serve to recruit or activate factors that directly inhibit RhoA activity. On the basis of our mass spectrometry results (Supplementary Data 1) we also identified ARHGAP11A (MP-GAP) and Anillin as p120 interaction partners. Indeed, we could precipitate p120-1A with either GFP-tagged MP-GAP or GFP-tagged Anillin from cell lysates (Supplementary Fig. 4c). Anillin is a scaffold protein that links RhoA to the actin machinery during cytokinesis^36^. Recent findings demonstrated that MP-GAP is a key GAP targeting RhoA activation during cytokinesis^16^ and showed that MP-GAP loss leads to a cytokinesis defect similar to p120 loss. We therefore focussed on MP-GAP and confirmed the observation that exogenous MP-GAP localized to the site of furrow ingression during anaphase (Supplementary Fig. 4d). This finding further underlines the significance of RhoA antagonists such as p120 and MP-GAP as modulators of rapid RhoA GTPase cycling at the equatorial cortex. During telophase, MP-GAP localized to the central spindle, specifically juxtaposed to the sites of phospho-MLC2 expression (Supplementary Fig. 4d). To visualize inactive RhoA, we employed phosphorylation of Serine 188 on RhoA (phospho-RhoA S188) as a surrogate readout, because this is an important event in the negative regulation of RhoA by protecting RhoA from degradation and inhibiting Rho-mediated stress fibre formation^37^,^38^,^39^. In anaphase, we found phospho-RhoA S188 localized to the plus ends of the microtubule spindle midzone, suggesting active translocation of phospho-RhoA S188 to the site of furrow ingression (Supplementary Fig. 4e). Indeed, this assumption was substantiated by the localization of phospho-RhoA S188 during telophase. Whereas the majority of total RhoA co-localized with p120 at the cell cortex, we observed only minimal phospho-RhoA expression at this site. Interestingly, most phospho-RhoA S188 expression appeared mutually exclusive to cortical RhoA (Supplementary Fig. 4e). These data suggest that during telophase, a pool of inactive RhoA is localized proximal to the sites of RhoA activation at the midbody, and as such might enable rapid fluxing of Rho activity; a process that is dependent on the presence of both positive and negative regulators near the equatorial cortex during cytokinesis. In this setting, p120 controls dominant suppression of actomyosin contraction and the formation of a balanced RhoA gradient through interaction with MKLP1 and the mitotic RhoGAP MP-GAP during late mitosis (Supplementary Fig. 5).

### Loss of p120 correlates with decreased patient survival

**Figure 6:**
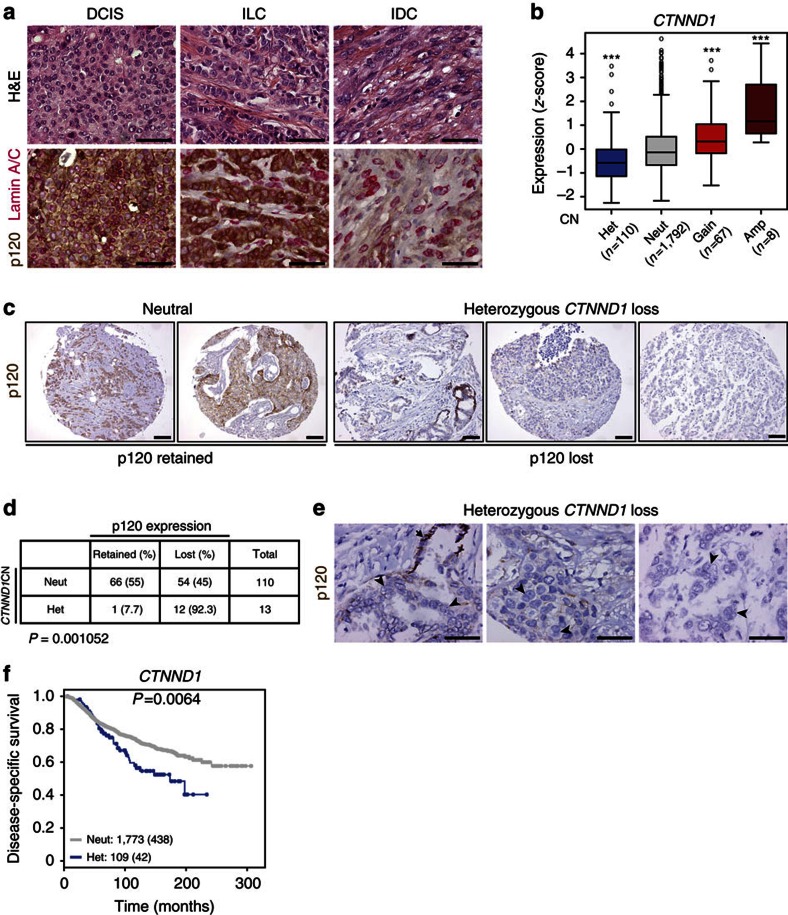
Heterozygous *CTNND1* loss correlates with loss of expression and decreased patient survival. (**a**) Immunohistochemistry (IHC) showing p120 and LaminA/C expression in human ductal carcinoma *in situ* (DCIS), invasive lobular carcinoma (ILC) and invasive ductal carcinoma (IDC). Scale bar, 50 μm. (**b**) Box plot showing the relation between *CTNND1* copy-number status (CN) and p120 mRNA expression. Shown are heterozygous (het), neutral (neut), gain and amplified (amp) CN status. Statistical significance was determined using pair-wise Tukey’s testing with the neutral copy-number group. ***P<0.001. (**c**)Tissue micro-array (TMA) cores of human breast cancer were stained and scored for p120. Shown are representative images of tumours neutral and heterozygous for *CTNND1* that were scored as positive (retained) or negative (lost) for p120 protein expression. Scale bar, 100 μm. (**d**) Quantification of p120 expression correlated to *CTNND1* copy number (CN) of TMA cores of human mammary carcinomas. Significance was determined using the Fisher’s exact test. (**e**) IHC showing p120 expression in human breast carcinomas with heterozygous genomic loss of *CTNND1*. Note the abnormal nuclear morphology in p120-deficient tumour cells (arrowheads) compared with p120 expressing cells (arrow). Scale bar, 50 μm. (**f**) Kaplan–Meier curve showing disease-specific survival of breast cancer patients displaying neutral *CTNND1* CN (grey; *n*=1,773) or heterozygous loss of *CTNND1* (blue; *n*=109). Statistical significance was determined using the log-rank test. Censored events are shown as dots and depicted between parentheses.

On the basis of our findings we examined breast cancer samples to correlate p120 loss with nuclear atypia and multinucleation and observed pronounced multinucleation in invasive ductal carcinoma (IDC) samples that displayed focal loss of p120 expression (Fig. 6a). In contrast, tumours expressing p120 such as ductal carcinoma *in situ* (DCIS) and ILC, did not display obvious nuclear abnormalities (Fig. 6a). Because mutation or promoter methylation of p120 is uncommon in cancer^22^, we determined the correlation between allelic *CTNND1* loss and p120 expression in a cohort of 1,977 invasive breast tumours. Interestingly, heterozygous loss was a relatively frequent event (110/1,977) that correlated with decreased mRNA expression (Fig. 6b and Supplementary Fig. 6a,b). We next analysed the corresponding samples by immunohistochemistry and observed significant enrichment for loss of p120 expression, indicating homozygous *CTNND1* inactivation (Fig. 6c,d). Importantly, we observed pronounced multinucleation and nuclear atypia in these p120-deficient tumours (Fig. 6e). Moreover, we detected a significant decrease (*P*=0.0064) in disease-specific survival in patients displaying heterozygous loss compared to patients with a neutral haplotype (Fig. 6f; Supplementary Fig. 6c,d). Altogether, these clinical data indicate that a high-grade and multinuclear phenotype correlates with p120-deficiency in breast cancer. Furthermore, our data are in agreement with the fact that p120 is not haplo-insufficient in tumour suppression, and suggest that heterozygous genomic inactivation predisposes to complete loss of p120 expression and subsequent adverse survival of cancer patients.

## Discussion

Cleavage furrow formation and ingression during cytokinesis depends on a balanced gradient of localized RhoA activity and subsequent actomyosin contractility^14^,^15^,^40^. Although the exact underlying mechanisms remain unclear, data from several labs including ours, support the notion that p120 can antagonize RhoA activity^29^,^33^,^34^,^35^. Regardless of whether p120 can directly modulate RhoA activity or impinges on RhoA activation indirectly through antagonizing molecules, our data clearly demonstrate that p120 is necessary to correctly position active RhoA and subsequent actomyosin contraction during cytokinesis. Due to its control over RhoA and by interacting with MKLP1 and MP-GAP, we envision that p120 might modulate rapid GTPase cycling by simultaneous binding to RhoA and the RhoGAP MP-GAP, whereas the interaction between p120 and MKLP1 provides the spatial signal that anchors RhoA cycling at the equatorial cortex during cytokinesis. Consistent with this hypothesis, we show that expression of the RhoA-binding mutant p120-1AΔ[622–628] was not sufficient to rescue the cytokinesis defects upon loss of p120. Interestingly, p120-1AΔ[622–628] was predominantly localized to the cytosol, suggesting that the association with RhoA promotes cortical positioning of p120 during mitosis and cytokinesis. In addition, reconstitutions with the N-terminal p120-1A truncation mutants p120-1AΔ[1–27] or p120-1AΔ[1–346] were not able to rescue the cytokinesis defects, although they still contained the domain that is responsible for RhoA inhibition. Cells expressing these mutants typically displayed a broadened or disturbed RhoA zone during cytokinesis, which is consistent with the notion that MKLP1 provides the spatial signal to ensure p120-dependent focused equatorial RhoA GTPase cycling. It has been proposed that MP-GAP may dominantly suppress lateral RhoA activation during cytokinesis^16^. Given the phenotypical, quantitive and spatiotemporal similarity in cytokinesis failure when depleting either p120 or MP-GAP, we think that regulation of p120-dependent RhoA inhibition may be controlled by MP-GAP. Because p120 depletion results in late cytokinesis failure and the fact that p120 also complexes with Anillin, we propose that p120 fulfils a scaffolding function between the actomyosin contractile ring and the central spindle by binding to MKLP1. Our results clearly indicate that p120 controls RhoA inactivation during cytokinesis, either through direct RhoA inhibition by unknown mechanisms, or by binding to MP-GAP. In doing so, p120 plays a decisive regulatory function by controlling formation of a balanced RhoA-GTP gradient by limiting the lateral diffusion of actomyosin contraction. In addition, the binding to MKLP1 suggests that p120 also might mediate spatial RhoA activation by Ect2, which is recruited to centralspindlin by MgcRacGAP during anaphase.

Our current data provide a rationale for the previous observation that p120-null keratinocytes displayed a high frequency of mitotic alterations^41^. We furthermore show that the function of p120 during cytokinesis is specific for p120 isoform 1A and is not restricted to the epithelial lineage. Moreover, p120 controls RhoA activity at the equatorial cortex independent of binding to classical cadherins, suggesting that different pools reside at specific locations to control designated cellular processes. Our data show that p120 is a general RhoA inhibitor at the cell membrane, but its association with key cytokinesis players renders it essential for local Rho inhibition during anaphase and telophase. For the regulation of actomyosin contraction during late mitosis, we envisage that cell-cycle-specific kinases might trigger post-translational modifications of the p120 N-terminus to regulate p120 spatio-temporally. The exact mechanisms and the kinases involved should be the centre of future research.

It has been become evident that p53 is essential in maintaining the diploid state by inducing a G1 arrest and/or apoptosis in binucleated tetraploid cells^42^,^43^,^44^. Recent data indicated that binucleated cells derived through chemically induced cytokinesis failure induced Hippo-dependent stabilization of p53 through inhibition of the E3 ligase MDM2 by LATS2 (ref. 45). These results probably explain the high penetrance of binucleation upon loss of p120, because we used conditional mouse models and cell lines that were either p53-null, p53 mutant, or overexpressed MDM2 (U2OS^46^). Interestingly, in human breast cancer loss of p120 is mainly observed in focal areas within the tumour^22^,^47^, suggesting that loss of p120 expression is a late event in tumour progression and is preceded by a first hit (for example, inactivation of p53). Supporting this are *in vivo* data from our lab and others that inactivation of p120 in the mammary gland is only tolerated in the context of p53 deficiency^22^,^48^. In contrast to the mammary gland, sole inactivation of p120 in the skin or the gastro-intestinal tract appears sufficient to induce hyper- proliferation and tumour formation^49^,^50^,^51^. Conditional p120 loss in keratinocytes was linked to mitotic defects and cancer development^41^,^50^, but it remains unclear whether p53 inactivation needs to precede p120 inactivation to allow the formation of multinucleated tumour cells in these organ types.

In conclusion, we provide an unprecedented and crucial function for p120 in regulation of cytokinesis, which is independent of its established role in cadherin-based cell–cell adhesion. Our data show that p120 plays a key regulatory role in maintaining a balanced and focused gradient of active RhoA through interactions with MKLP1, MP-GAP and Anillin during cytokinesis. Loss of p120 expression, a common feature of invasive cancers, therefore not only destabilizes cadherin-dependent cell–cell adhesion, but also induces multinucleation and CIN by introducing RhoA-dependent cytokinesis defects.

## Methods

### Origin of animal models and cell lines

Generation of all animal models and mouse mammary cell lines described in this study have been previously reported^22^,^23^,^24^,^35^. U2OS, T47D and HeLa cells were obtained from ATCC (#HTB-96, #HTB-133 and #CCL-2, respectively). All cell lines were cultured in DMEM/F12 supplemented with 6% tetracyclin-free FCS (#SH3007103; HyClone), 2 mM glutamine (#BE17-605E; Lonza), 100 U ml^−1^ penicillin and 10 μg ml^−1^ streptomycin. For mouse cell lines the culture medium was additionally supplemented with 5ng·ml^−1^ EGF and 5 μg·ml^−1^ insulin (#I9278; Sigma). For induction of shRNA expression cells were incubated with 1 μg·ml^−1^ doxycyclin for 4 days. For synchronization, U2OS cells were incubated with thymidine (2.5 mM in culture medium) for 20 h to arrest cells in S-phase. Afterwards, cells were washed multiple times with warm medium, released for 4 h and incubated with nocodazole (0.83 μM in culture medium) for 14 h to arrest cells in pro-metaphase. Cells were again washed multiple times and fixated at different time points to enrich for cells in different stages of mitosis and cytokinesis.

### Immunohistochemistry

Tissues were isolated and fixed in 4% formalin. Tissues were dehydrated, cut into 4 μm sections, and stained with hematoxylin and eosin. For immunohistochemical stainings, fixed sections were rehydrated and incubated with primary antibodies. The following primary antibodies were used: mouse anti-p120 (1:500; #610134; BD Biosciences) and rabbit anti-Lamin A (1:200; #L1293; Sigma). For mouse tissue, endogenous peroxidases were blocked with 3% H_2_O_2_ and biotin-conjugated secondary antibodies were used, followed by incubation with HRP-conjugated streptavidin–biotin complex (DAKO). Substrate was developed with DAB (DAKO). For human tissue, stainings were performed using a Ventana BenchMark ULTRA (Roche) and detections were performed using DAB (DAKO) and NovaRED (#SK-4805; Vector Labs). All scoring was done blinded to patient characteristics and results of other staining by two independent observers. Expression of p120 was scored as membranous and/or cytosolic (0 (lowest), 1, 2 and 3(highest)). Expression of p120 was considered lost when the membranous score and the cytosolic score ≤1. Imaging was performed using a Nikon Eclipse E800 microscope mounted with a Nikon digital camera DXM1200.

### Immunofluorescence microscopy

Cells were allowed to adhere to glass coverslips overnight and subsequently fixed with 10% trichloroacetic acid (TCA; for staining of RhoA) or 4% paraformaldehyde. After TCA fixation, cells were washed three times with 30 mM glycine in PBS for 5 min (ref. 52), and permeabilized using 0.25% Triton-X100 in PBS for 5 min at room temperature. Samples were blocked with 5% milk in PBS (for RhoA stainings) or 5% BSA in PBS for 1 h. Samples were incubated with primary antibodies overnight at 4 °C. The following primary antibodies were used: rabbit anti-LaminA/C (1:1,000, #L1293; Sigma) mouse anti-p120 (1:500; #610134; BD Biosciences), mouse anti-p120-TRITC (1:300; #610137; BD Biosciences), mouse anti-p120 6H11 (1:500; Santa Cruz Biotechnology), rat anti-GFP mouse anti-RhoA 26C4 (1:200; sc-418; Santa Cruz Biotechnology), rat anti-E-cadherin (1:200; #43254; Sigma), rabbit anti-MKLP1/KIF23 (1:200; sc-857; Santa Cruz Biotechnology), rabbit anti-Ect2 (1:100; #07-1364; Merck), mouse anti-alpha Tubulin (1:10,000; T5168; Sigma-Aldrich), rat anti-alpha Tubulin YL1/2 (1:500; MA1-80017; Thermo Scientific), rat anti-GFP (1:2000; 3H9 Chromotek) and rabbit anti-γ Tubulin (1:500; T5192; Sigma-Aldrich). For detection, samples were incubated with secondary antibodies in blocking buffer for 1 h at room temperature. The following secondary antibodies were used: (1:600; Invitrogen): goat anti-mouse Alexa488 (#A11029), goat anti-rabbit Alexa488 (#A11034), goat anti-mouse Alexa568 (#A11031), goat anti-rabbit Alexa568 (#A11036), goat anti-rabbit Alexa647 (#A21245) and goat anti-rat Alexa647 (#A21247). Afterwards, cells were incubated with Alexa633-phalloidin to visualize F-actin (1:300; #A22284; Life Technologies) or DAPI to stain DNA, washed and subsequently mounted using ImmunoMount (#9990402; Thermo Scientific). Imaging was performed on a LSM700 confocal microscope (Zeiss). Image processing was performed using ImageJ and Photoshop (Adobe).

### Quantification of bi/multinucleation and centrosomes

For quantification of binucleation and multinucleation, cells were allowed to adhere to glass coverslips overnight, fixated with either ice-cold methanol and subsequent stained for α-Tubulin and DNA (DAPI) for quantification of bi- and multinucleation or fixated with 4% paraformaldehyde and stained for γ-Tubulin, α-Tubulin and DNA (DAPI) for quantification of centrosome numbers. Multinucleation was defined as >2 nuclei per cell. For both experiments, random fields (× 63) were imaged with a LSM700 (Zeiss) and statistical significance was determined using the χ^2^-test.

### mRNA expression, copy number and survival data METABRIC

DNA and RNA from matched pairs were extracted for 1980 tumours^53^. Copy-number analysis was performed using the Affymetrix SNP 6.0 platform. These arrays were pre-processed and normalized using CRMAv2 (ref. 54) method from the aroma.affymetrix R package. For each of them, allelic-crosstalk calibration, probe sequence effects normalization, probe-level summarization and PCR fragment length normalization were performed. Then the array intensities were normalized against a pool of 473 normal samples for those that had no matched normal or against their matched normal when available. The log-ratios were segmented using the CBS algorithm^55^ from the DNAcopy Bioconductor package. Finally, copy-number callings into five groups (homozygous deletion, heterozygous deletion, neutral copy number, gain and amplification), were made using thresholds based on the variability of each sample and their proportion of normal contamination. RNA analysis was performed using Illumina HT-12 v3 platform and analysed with the beadarray package^56^. BASH^57^ algorithm was employed to correct for spatial artefacts. Bead-level data was summarized and a selection of suitable probes based on their quality was done using the re-annotation of the Illumina HT-12v3 platform described in ref. 58. The samples were classified into the five breast cancer subtypes using PAM50 (ref. 59). Kaplan–Meier estimates and log-rank tests were obtained using the survival R package. Comparison of expression levels and copy-number states was performed fitting a linear model and then computing simultaneous testing using Tukey’s method from the multicomp R package^60^.

### COBRA-FISH

For gross analysis of structural variation, COBRA-FISH analysis was performed as described before (detailed protocol available in reference)^61^.

### Constructs

Cloning of the doxycycline-inducible shRNA constructs directed against mouse and human p120 and generation of the non-targetable p120 cDNA expression construct (pLV.CMV.p120.IRES.puro) were previously described^22^. All p120 mutants were generated by PCR-directed cloning, using the non-targetable p120 cDNA in pEGFP-C1 as template. PCR was performed using Phusion DNA polymerase (#M0530S;NEB) and corresponding primers (Table 1). Amplicons were phosphorylated with T4 polynucleotide kinase (#M0202L; NEB) and ligated. Afterwards, all GFP-tagged constructs were amplified from the vector with primers containing AscI and NheI sites, digested and ligated in pLV.CMV.IRES.puro. All constructs were sequence verified. Constructs used for pulldown experiments (RC-CMV-p120-1A, -3A, -4A and ΔARM(1–10)) were a kind gift from P. Anastasiadis (Mayo Clinic, Jacksonville, FL), and were previously described^62^. GFP-tagged MKLP1 in pEGFP-C1 was a kind gift from A. Akhmanova (Utrecht University, The Netherlands) and GFP-tagged Anillin was obtained from Addgene (pEGFP-Anillin; #68027). GFP-tagged MP-GAP (ARHGAP11A) was generated by PCR using Phusion polymerase (Thermo Fischer Scientific) from a cDNA library derived from U2OS mRNA (kind gift from Susanne Lens) using the following primers (5′-GCTACTCGAGCTATGTGGGATCAGAGGCTG-3′ and 5′-TGCAGGATCCTTACAAATCTACAGGTTTACTTGTTGG-3′). The resulting amplicon, flanked by EcoRI and BamHI sites, was cloned into pEGFP-C1 (Clontech) to generate GFP-tagged MP-GAP.

### Time-lapse imaging

To visualize mitosis and cytokinesis, U2OS cells were transduced with Baculovirus expressing H2B-mCherry in combination with either GFP-α-tubulin (kind gifts from M. Hadders and S. Lens, University Medical Center Utrecht, Utrecht, The Netherlands) or GFP-actin (CellLight; C10582; Life Technologies). Cells were imaged with a Personal Deltavision (GE Healthcare) in Leibovitz medium (Gibco) supplemented as described in previous sections. Images were processed with ImageJ.

### FRET microscopy and image processing

The RaichuEV-RhoA biosensor was a kind gift from Professor M. Matsuda, Kyoto University, Japan. U2OS cells were transfected with the probe containing the PiggyBac transposon system (Wellcome Trust Sanger Institute, Hinxton, UK) using Lipofectamine 2,000 Transfection Reagent (Life Technologies) according to manufacturer’s instructions. Cells were seeded on 35 mm glass-base dishes at a density of 1.5 × 10^4^ and transfected with the H2B-mCherry baculovirus overnight to allow visualization of the nucleus. FRET images were acquired using a Zeiss 710 inverted microscope equipped with 458 nm argon laser and 561 nm DPSS laser through an MBS 458/561 filter. Emission light of the biosensor was separated by beam splitters into 462–511 nm for Turquoise-GL, 521–559 nm for FRET and 602–697 nm for mCherry. Stacked images of seven sections (0.85 μm distance) were acquired every 5 min to capture FRET information in 3D for up to 24 h. Using Metamorph software, sectioned images were made into sum stacks and background subtracted before generating a ratio image (YFP/CFP).

### siRNA-mediated MKLP1 depletion

U2OS cells were transfected with siRNAs (final concentration 20 μM) directed against MKLP1 (5′-CGACAUAACUUACGACAAAUU-3′) or luciferase (5′-CGUACGCGGAAUACUUCGA-3′). In short, siRNAs were mixed with 1.5 μl HiPerfect Transfection Reagent (#301705; Qiagen) in 100 μl OptiMEM and incubated at room temperature for 30 min. The reaction mixture was added to the cell suspension in a 24-well plate (Costar) containing a 12 mm glass coverslip. The next day, cells were incubated with thymidine at a final concentration of 2.5 mM for 24 h. Afterwards, cells were released through multiple washes and fixed ∼8 h later to obtain cells in anaphase/telophase.

### Mass spectrometry

Preparation of samples and mass spectrometry were performed as previously described^35^. In summary, protein bands were stained and visualized using SYPRO Ruby (Invitrogen). Peptides were extracted with 10% formic acid (BDH) and subjected to LC–tandem MS analysis (1,100 HPLC system; Agilent Technologies) connected to an LTQ Linear Ion Trap Mass Spectrometer combined with an Orbitrap (ThermoFisher). The International Protein Index (IPI) mouse database (http://www.ebi.ac.uk/IPI/) was used as a reference using Mascot software (version 2.2.0; Matrix Science). Peptides with mascot scores greater than 30 were considered for further analysis.

### Anti-GFP immunoprecipitations and western blotting

293T cells plated in a 10 cm culture dish were transfected using XtremeGene9 (#06365809001; Roche) and 2 μg GFP-MKLP1 and 6 μg of either p120-1A, p120-3A, p120-4A, p120ΔARM or empty vector. GFP-Trap anti-GFP beads (#gta-20; Chromotek) were used to perform the GFP-MKLP1, GFP-Anillin and GFP-MP-GAP co-immunoprecipitations. Western blotting was performed using the following primary antibodies: mouse anti-p120 (1:2,000; Clone 98/pp120; Becton Dickinson), rabbit anti-p120 (1:500; H90/sc-13957; Santa Cruz Biotechnology), rabbit anti-GFP (1:500; sc-8334, Santa Cruz Biotechnology), mouse anti-RhoA (1:300; 26C4/sc-418; Santa Cruz Biotechnology) and goat anti-Akt (1:1,000, C-20/sc-1618; Santa Cruz Biotechnology). Uncropped blots are supplied in Supplementary Fig. 7.

### Data availability

All relevant data are available from the authors on request and/or are included with the manuscript (as figure source data or Supplementary Information files).

## Additional information

**How to cite this article:** van de Ven, R. A. H. *et al*. p120-catenin prevents multinucleation through control of MKLP1-dependent RhoA activity during cytokinesis. *Nat. Commun.* **7,** 13874 doi: 10.1038/ncomms13874 (2016).

**Publisher’s note**: Springer Nature remains neutral with regard to jurisdictional claims in published maps and institutional affiliations.

## Supplementary Material

Supplementary Information Supplementary Figures and Supplementary Tables

Supplementary Movie 1 Loss of p120 induces severe and continuous membrane deformation during cytokinesis. Time-lapse imaging of non-treated (Supplementary Movie 1) and dox-treated (Supplementary Movie 2) U2OS p120-iKD cells transduced with H2B-mCherry. Shown are merges of 100X images of DIC and mCherry (red) channels. Bar = 10 μm.

Supplementary Movie 2 Loss of p120 induces severe and continuous membrane deformation during cytokinesis. Time-lapse imaging of non-treated (Supplementary Movie 1) and dox-treated (Supplementary Movie 2) U2OS p120-iKD cells transduced with H2B-mCherry. Shown are merges of 100X images of DIC and mCherry (red) channels. Bar = 10 μm.

Supplementary Movie 3 Loss of p120 induces oscillating waves of RhoA activity resulting in cytokinesis failure. Time-lapse imaging of non-treated (Supplementary Movie 3) and dox-treated (Supplementary Movie 4) U2OS p120-iKD cells transduced with EV-Raichu RhoA-specific FRET probe. Shown are 63X images of YFP/CFP ratio images and H2B-mCherry (white). Bar = 20 μm.

Supplementary Movie 4 Loss of p120 induces oscillating waves of RhoA activity resulting in cytokinesis failure. Time-lapse imaging of non-treated (Supplementary Movie 3) and dox-treated (Supplementary Movie 4) U2OS p120-iKD cells transduced with EV-Raichu RhoA-specific FRET probe. Shown are 63X images of YFP/CFP ratio images and H2B-mCherry (white). Bar = 20 μm.

Supplementary Movie 5 Loss of p120 induces aberrant accumulation of F-actin during cytokinesis. Time-lapse imaging of non-treated (Supplementary Movie 5) and dox-treated (Supplementary Movie 6) U2OS p120-iKD cells transduced with GFP-actin and H2B-mCherry. Shown are merges of 100X images of GFP (green) and mCherry (red). Bar = 10 μm.

Supplementary Movie 6 Loss of p120 induces aberrant accumulation of F-actin during cytokinesis. Time-lapse imaging of non-treated (Supplementary Movie 5) and dox-treated (Supplementary Movie 6) U2OS p120-iKD cells transduced with GFP-actin and H2B-mCherry. Shown are merges of 100X images of GFP (green) and mCherry (red). Bar = 10 μm.

Supplementary Dataset 1 p120-specific interaction partners in mILC-1.

## Figures and Tables

**Table 1:** primers used to clone p120-1A constructs.

**Construct**	**Forward primer**	**Reverse primer**
p120-1A K401M	5′-ATGACTGACGTGCGGAAGC-3′	5′-CACCTTGTCATTGCGGTAGC-3′
p120-1AΔ[622–628]	5′-CCTACAGAGGATCCAGCAAATGATAC-3′	5′-GGCTCCAAAGCAACTGGC-3′
p120-1AΔ[1–27]	5′-CGGGCGCTGGAAGAGG-3′	5′-GGTGGCGGAATTCGAAGC-3′
p120-1AΔ[1–347]	5′-TTAGCAAGCTTGGATAGTTTGCG-3′	5′-GGTGGCGGAATTCGAAGC-3′
