# Corrigendum: p120-catenin prevents multinucleation through control of MKLP1-dependent RhoA activity during cytokinesis

**DOI:** 10.1038/ncomms16030

**Published:** 2017-09-06

**Authors:** Robert A. H. van de Ven, Jolien S. de Groot, Danielle Park, Robert van Domselaar, Danielle de Jong, Karoly Szuhai, Elsken van der Wall, Oscar M. Rueda, H. Raza Ali, Carlos Caldas, Paul J. van Diest, Martin W. Hetzer, Erik Sahai, Patrick W. B. Derksen

Nature Communications 7: Article number: 13874 ; DOI: 10.1038/ncomms13874 (2016); Published 12 22 2016; Updated 09 06 2017

The financial support for this Article was not fully acknowledged. The Acknowledgements should have included the National Institutes of Health grants R01GM098749 and National Institutes of Health Transformative Research Award R01NS096786.

