# Supplementary material for: p120-catenin prevents multinucleation through control of MKLP1-dependent RhoA activity during cytokinesis
Source: Nat Commun. 2016 Dec 22;7:13874. doi: 10.1038/ncomms13874 (PMC5192218; doi:10.1038/ncomms13874)
Supplement: Supplementary Information — Supplementary Figures and Supplementary Tables [file ncomms13874-s1.pdf]

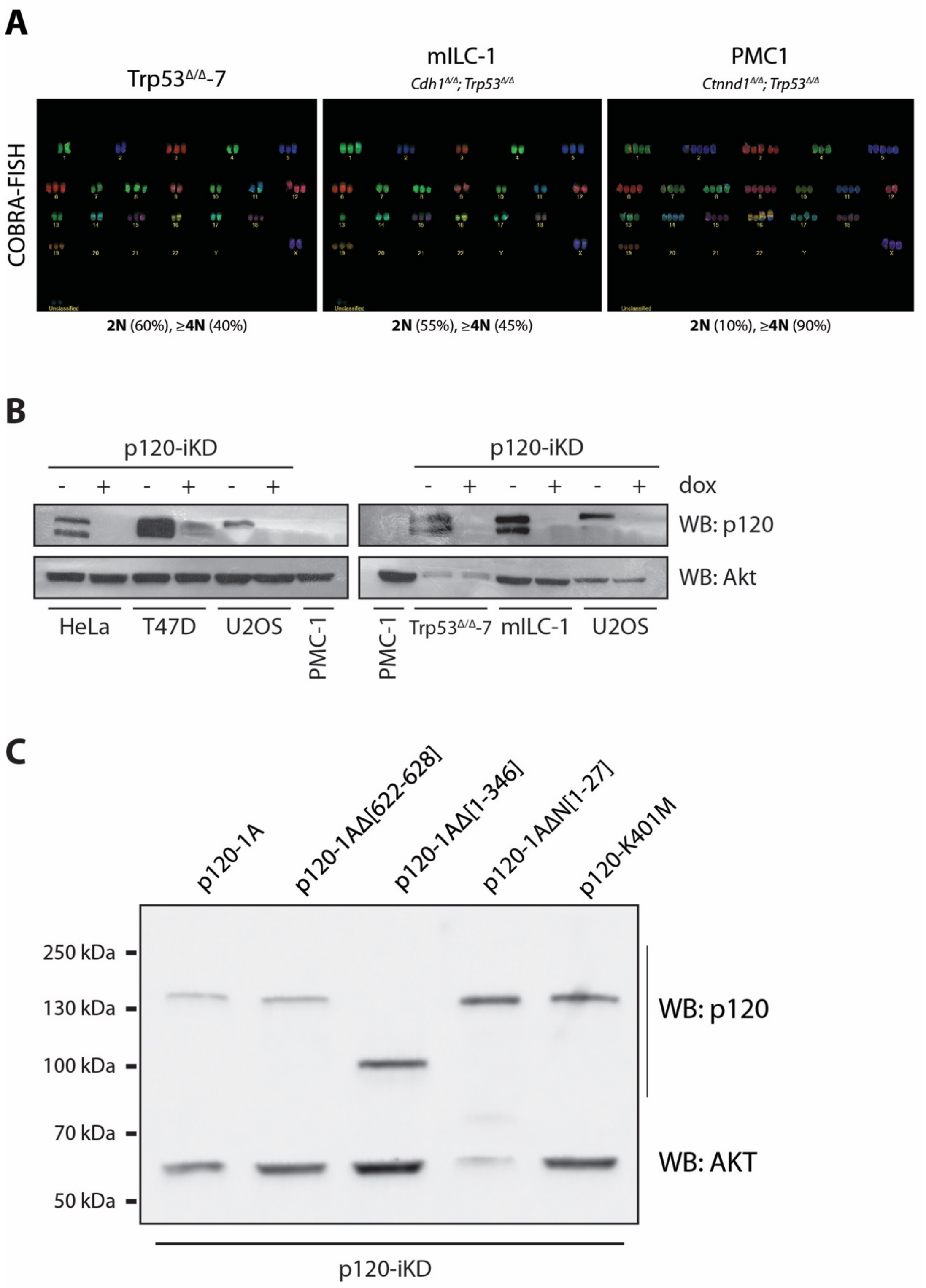
A
Trp53Δ/Δ-7
mILC-1
Cdh1Δ/Δ; Trp53Δ/Δ
PMC1
Ctnnd1Δ/Δ; Trp53Δ/Δ
COBRA-FISH
2N (60%), ≥4N (40%)
2N (55%), ≥4N (45%)
2N (10%), ≥4N (90%)
B
p120-iKD
p120-iKD
dox
WB: p120
WB: Akt
HeLa
T47D
U2OS
PMC-1
PMC-1
Trp53Δ/Δ-7
mILC-1
U2OS
C
p120-1A
p120-1AΔ[622-628]
p120-1AΔ[1-346]
p120-1AΔN[1-27]
p120-K401M
250 kDa
130 kDa
100 kDa
70 kDa
50 kDa
WB: p120
WB: AKT
p120-iKD

Supplementary Figure 1: p120 depletion in PMC-1 and p120-iKD cell lines

A Multi colour COBRA-FISH analysis of chromosomal content in $Trp53^{\Delta/\Delta}$-7, mILC-1 and PMC-1 cell lines. Quantification of ploidy in analyzed cells is displayed (n=20; more details in Supplementary Table 1). **B** Western blot showing p120 depletion upon dox treatment in a range of different p120-iKD human and mouse cancer cell lines. Akt was used as a loading control. **C** Reconstitution of p120 variants in p120 iKD U2OS cells. Shown is a western blot probed for p120. AKT served as a loading control.

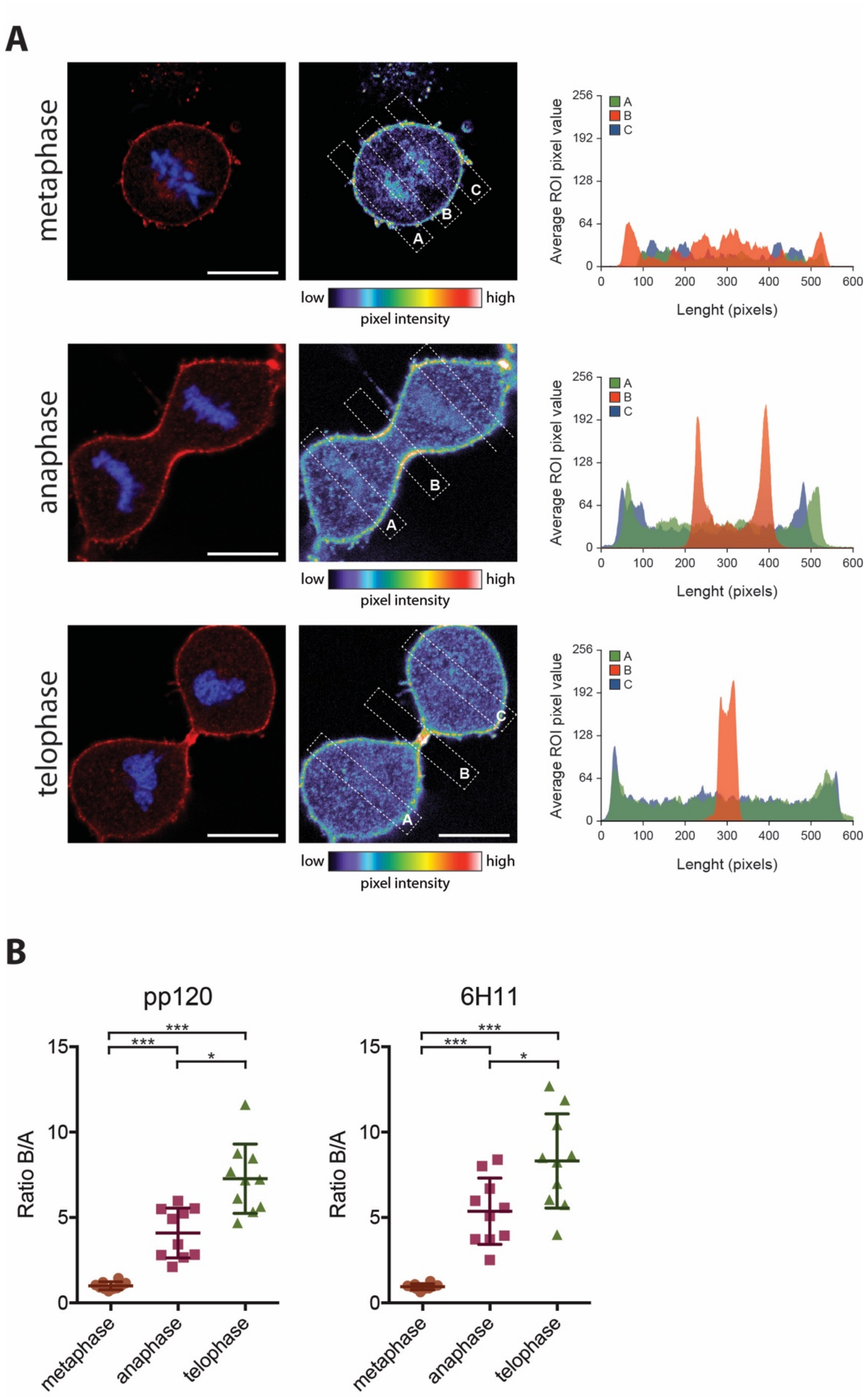
A
metaphase
anaphase
telophase
A
B
C
low
high
pixel intensity
Average ROI pixel value
Lenght (pixels)
0
64
128
192
256
0
100
200
300
400
500
600
B
pp120
6H11
Ratio B/A
***
*
0
5
10
15
metaphase
anaphase
telophase

Supplementary Figure 2: p120 is enriched at the cleavage furrow

A IF for p120 using the monoclonal antibody pp120 (clone 99; pan p120) on U2OS cells. Shown are the fluorescence signals (left panels) and a pseudo-coloured image that depicts the three regions used for pixel quantification. Pixel intensity scale is shown below the images. The right hand graphs show the quantified signal of the three regions. Boxes A and C represent two peripheral cortex regions, box B represents the ingression/cleavage furrow. Note the marked accumulation of the p120 signal (p120) in box B. **B** Quantification of the p120-specific fluorescence signal derived from mitotic U2OS cells using two independent anti-p120 antibodies namely pp120 (pan p120) and 6H11 (p120-1A specific). Shown is the ratio in maximal fluorescence signal between box A and box B as described in (A). Note the marked increase in p120 signal specifically in anaphase and telophase cells. Ten cells were quantified for each cell cycle stages per antibody. Statistical significance was determined using the student's t-test. * = $p<0.05$, ** = $p<0.01$, *** = $p<0.001$.

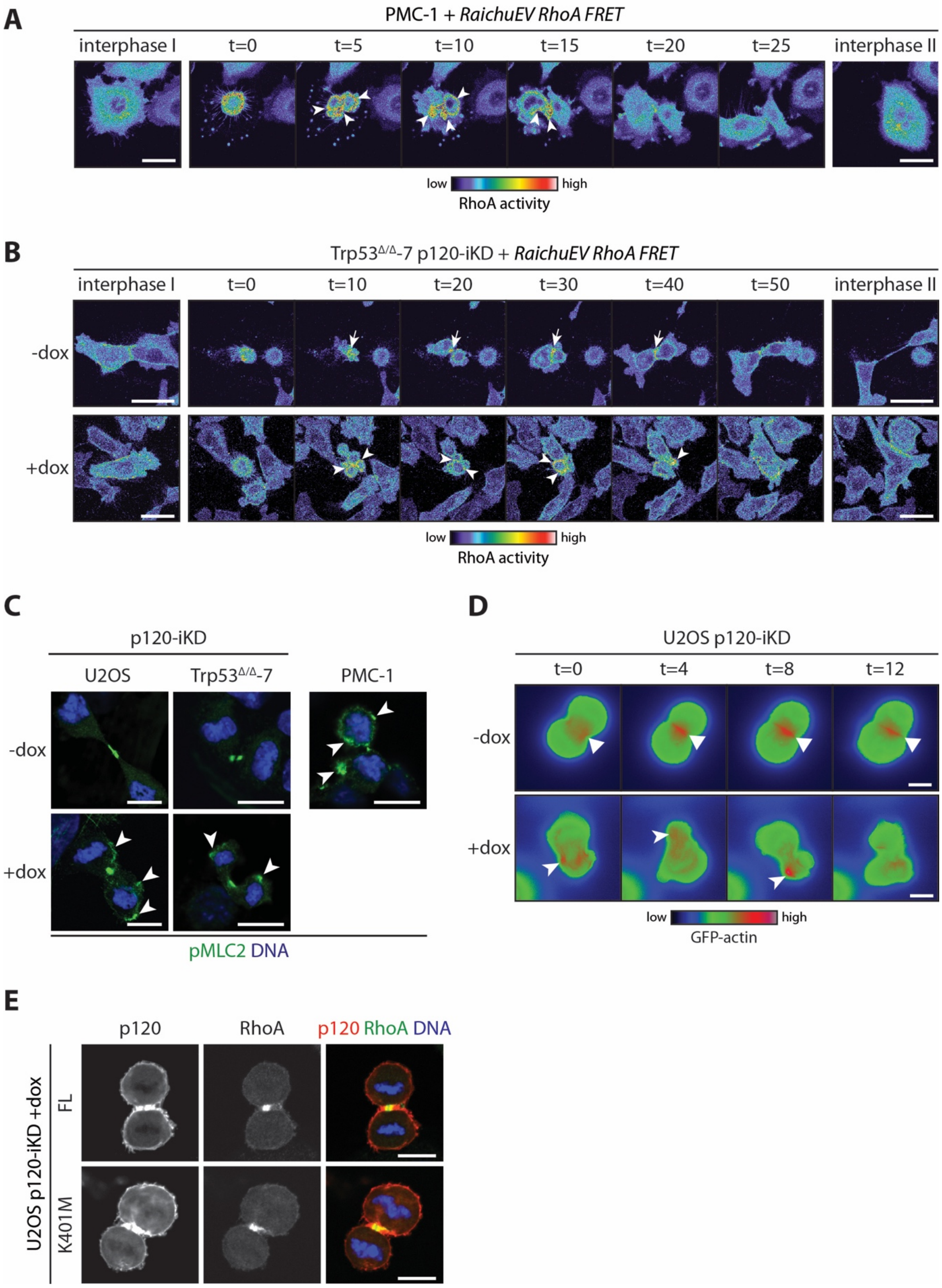
A
PMC-1 + RaichuEV RhoA FRET
interphase I
t=0
t=5
t=10
t=15
t=20
t=25
interphase II
low
high
RhoA activity
B
Trp53Δ/Δ-7 p120-iKD + RaichuEV RhoA FRET
interphase I
t=0
t=10
t=20
t=30
t=40
t=50
interphase II
-dox
+dox
low
high
RhoA activity
C
p120-iKD
U2OS
Trp53Δ/Δ-7
PMC-1
-dox
+dox
pMLC2 DNA
D
U2OS p120-iKD
t=0
t=4
t=8
t=12
-dox
+dox
low
high
GFP-actin
E
p120
RhoA
p120 RhoA DNA
U2OS p120-iKD +dox
FL
K401M

Supplementary Figure 3: Loss of p120 induces aberrant RhoA activity during cytokinesis

A and **B** Time-lapse stills of PMC-1 (A) and Trp53$^{\Delta/\Delta}$-7 p120-iKD (B) cells expressing the RaichuEV RhoA FRET probe. Note the mislocalised RhoA activity during anaphase (arrowheads). Bar = 20 µm. **C** IF staining for phosphorylated myosin light chain 2 (pMLC2) in PMC-1, U2OS p120-iKD and Trp53$^{\Delta/\Delta}$-7 p120-iKD cells. Note the aberrant localisation of pMLC2 in p120 depleted cells (arrows). Bar = 10 µm. **D** Pseudo colour time-lapse images of control and dox-treated U2OS p120-iKD transduced with GFP-actin. Shown are 100X stills. Bar = 10 µm. **E** IF staining for p120 and RhoA in dox-treated U2OS p120-iKD cells reconstituted with FL p120-1A and p120-1A K401M. Bar = 10 µm.

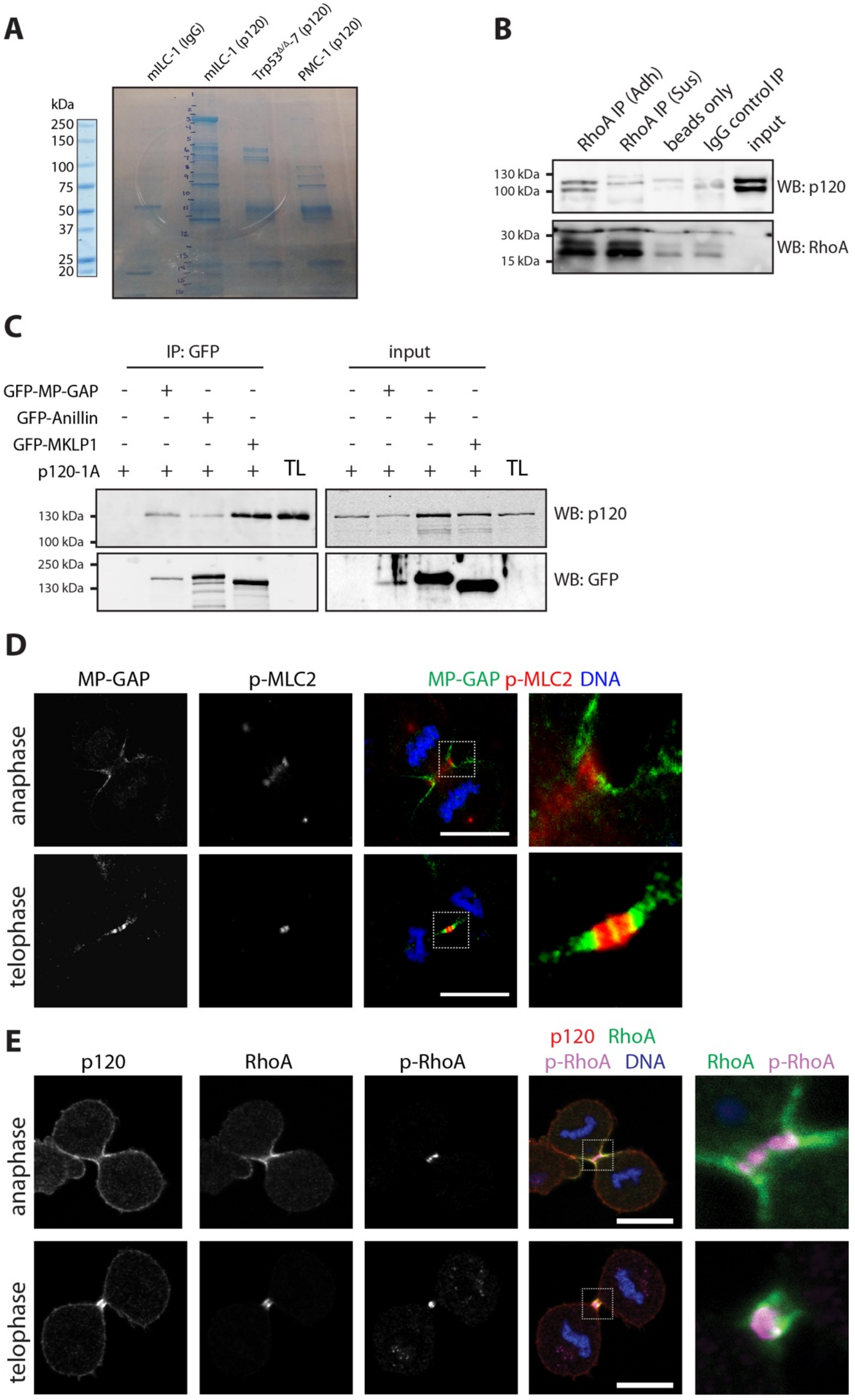
A
mILC-1 (IgG)
mILC-1 (p120)
Trp53Δ/Δ-7 (p120)
PMC-1 (p120)
kDa
250
150
100
75
50
37
25
20
B
RhoA IP (Adh)
RhoA IP (Sus)
beads only
IgG control IP
input
130 kDa
100 kDa
WB: p120
30 kDa
15 kDa
WB: RhoA
C
IP: GFP
input
GFP-MP-GAP
GFP-Anillin
GFP-MKLP1
p120-1A
TL
130 kDa
100 kDa
WB: p120
250 kDa
130 kDa
WB: GFP
D
MP-GAP
p-MLC2
MP-GAP p-MLC2 DNA
anaphase
telophase
E
p120
RhoA
p-RhoA
p120 RhoA p-RhoA DNA
RhoA p-RhoA
anaphase
telophase

Supplementary Figure 4: p120 forms a complex with RhoA, MP-GAP and Anillin at the interphase of actomyosin contraction during cytokinesis.

A Coomassie-stained gel showing the different bands that were excised and used in mass spectrometry experiments. Cell lysates from either mILC-1 (lane 2) or PMC-1 (lane 4; negative control) were subjected to anti-p120 co-immunoprecipitations and subsequent mass spectrometry. Control IgG co-immunoprecipitations on mILC-1 (lane 1) was used as an additional negative control. Lane 3 shows a anti-p120 co-immunoprecipitation on Trp53$^{\Delta/\Delta}$-7. **B** Asynchronous mILC-1 cells from adherent (Adh) or anchorage-independent (Sus) were harvested and RhoA was extracted from the lysates. Shown are the resulting western blots probed for p120 (upper panel) and RhoA (lower panels). We were not able to detect RhoA in the input lane probably due to the relative low expression compared to the levels precipitated from the same sample. **C** GFP-tagged cDNAs for MP-GAP, Anillin or MKLP1 were expressed in 293T cells together with p120-1A, and immunoprecipitated using GFP-Traps. IPs (left panels) and total lysates (right panels) were western blotted and probed for p120 (top panels) or GFP (bottom panels). TL represents a control total lysate from U2OS. **D** IF for MP-GAP and phospho-MLC2 (p-MLC2) in anaphase and telophase U2OS cells. Note the mutually exclusive localisation of MP-GAP, and p-MLC2 at the equatorial cortex (top panels) and the midbody (bottom panels). Right panels show representative magnifications of representative areas denoted by the dotted squares. Bar = 10 µm. **E** IF for p120, RhoA and RhoA-Ser188 (p-RhoA) in anaphase and telophase U2OS cells. Right panels show magnifications of representative areas denoted by the dotted squares. Bar = 10 µm. Note the marked pool of p-RhoA (pink) in close vicinity to cortical RhoA (green).

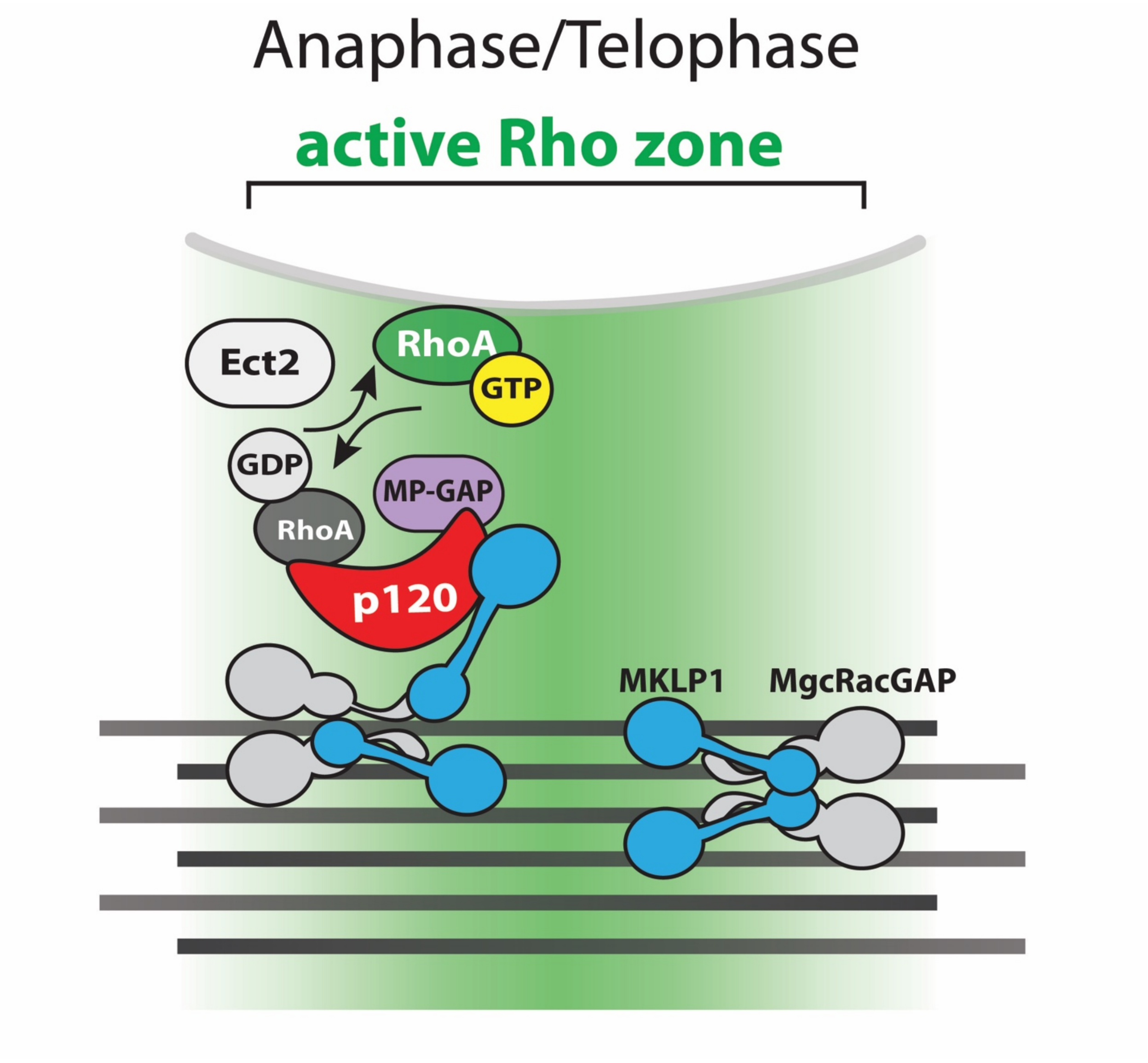

Supplementary Figure 5: Proposed model for the p120-dependent regulation of cytokinesis

During anaphase, p120 associates with the active RhoA zone at the equatorial cortex. Here, it regulates spatial RhoA activity through binding to the centralspindlin component MKLP1 that brings RhoA in close proximity to the RhoGEF Ect2 that is recruited to the other centralspindlin member, MgcRacGAP. The ability of p120 to directly bind GDP-bound RhoA will contribute to rapid GTPase cycling of RhoA, a process that limits lateral diffusion and restricts the active RhoA zone, which assures focused actomyosin contractility and correct cleavage furrow ingression.

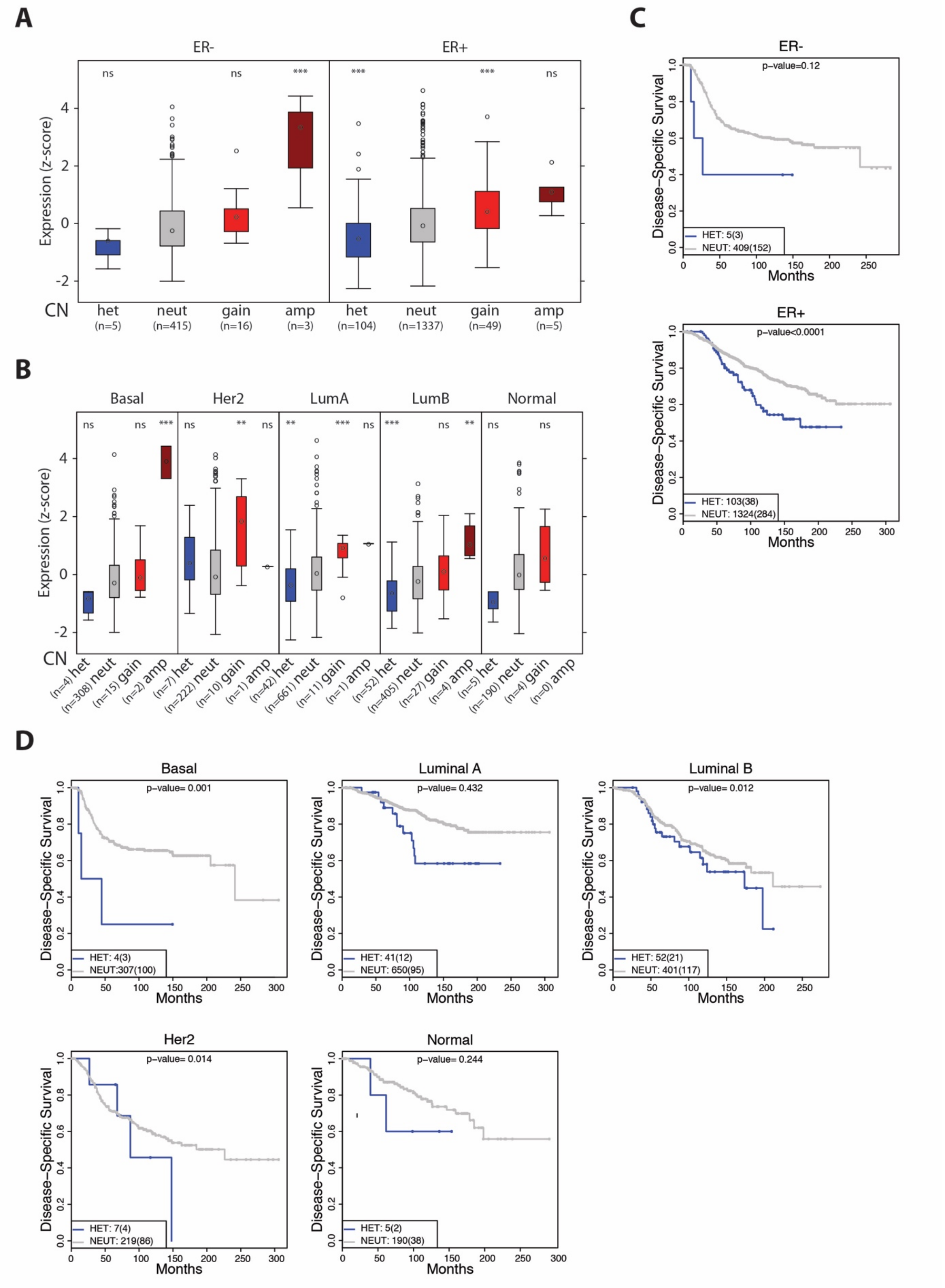

A
ER-
ER+
Expression (z-score)
CN
het
(n=5)
neut
(n=415)
gain
(n=16)
amp
(n=3)
het
(n=104)
neut
(n=1337)
gain
(n=49)
amp
(n=5)
B
Basal
Her2
LumA
LumB
Normal
Expression (z-score)
CN
(n=4) het
(n=308) neut
(n=15) gain
(n=2) amp
(n=7) het
(n=222) neut
(n=10) gain
(n=1) amp
(n=42) het
(n=661) neut
(n=11) gain
(n=1) amp
(n=52) het
(n=405) neut
(n=27) gain
(n=4) amp
(n=5) het
(n=190) neut
(n=4) gain
(n=0) amp
C
ER-
p-value=0.12
Disease-Specific Survival
HET: 5(3)
NEUT: 409(152)
Months
ER+
p-value<0.0001
Disease-Specific Survival
HET: 103(38)
NEUT: 1324(284)
Months
D
Basal
p-value= 0.001
Disease-Specific Survival
HET: 4(3)
NEUT:307(100)
Months
Luminal A
p-value= 0.432
Disease-Specific Survival
HET: 41(12)
NEUT: 650(95)
Months
Luminal B
p-value= 0.012
Disease-Specific Survival
HET: 52(21)
NEUT: 401(117)
Months
Her2
p-value= 0.014
Disease-Specific Survival
HET: 7(4)
NEUT: 219(86)
Months
Normal
p-value= 0.244
Disease-Specific Survival
HET: 5(2)
NEUT: 190(38)
Months

Supplementary Figure 6: *CTNND1* copy number status, expression and survival data in breast cancer patients

A and **B** Relation between *CTNND1* expression and copy number (CN) status in breast cancer patients subcategorised into (A) ER- and ER+ and (B) basal/Her2/LumA/LumB/normal subtypes. Shown are heterozygous (het), neutral (neut), gain and amplified (amp) CN status. Statistical significance was determined using pair-wise Tukey's testing with the neutral haplotype. * $p<0.05$, ** = $p<0.005$ and *** = $p<0.001$. **C** and **D** Kaplan-Meier curves showing disease-specific survival of breast cancer patients displaying neutral *CTNND1* CN (NEUT; gray) or heterozygous loss of *CTNND1* (HET; blue) subcategorised into (C) ER- and ER+ and (D) basal/Her2/LumA/LumB/normal. Significance was determined using the log rank test. Censored events are shown as dots and depicted between parentheses.

Blots from Figure 5D - Complete blots and blot strips are already depicted in figure

Blots from Supplementary Figure 1C:

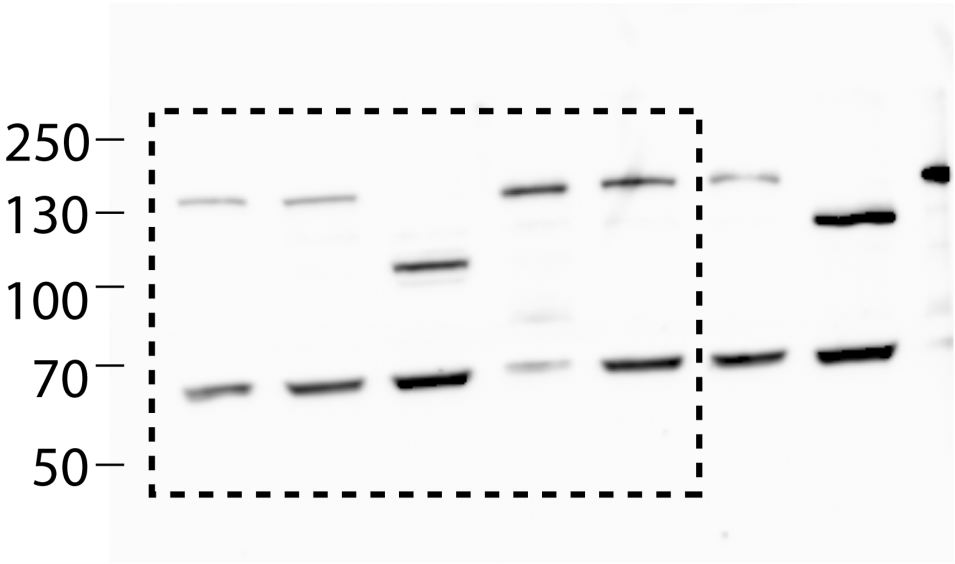

Blots from Supplementary Figure 4A:

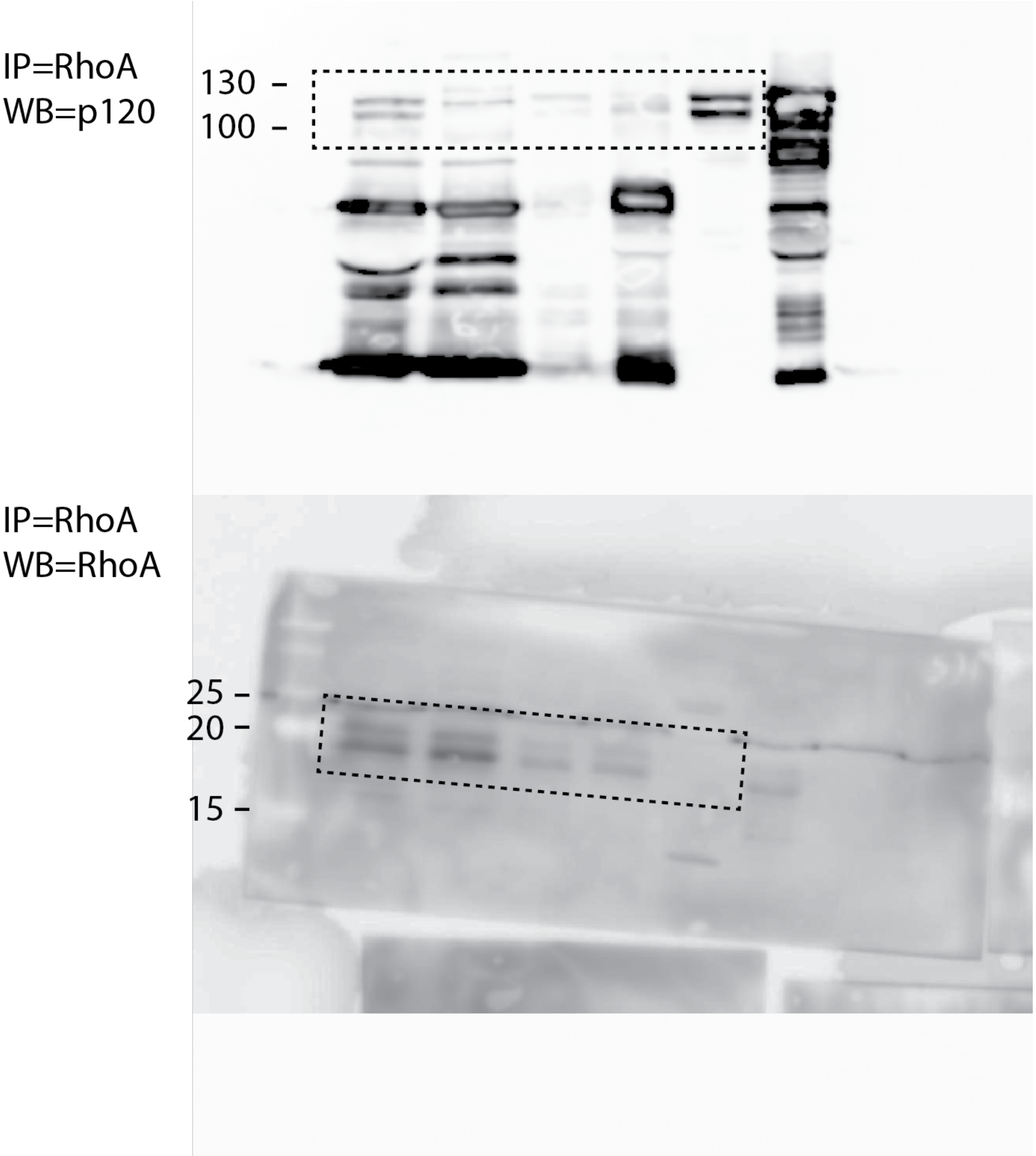

Blots from Supplementary Figure 4B:

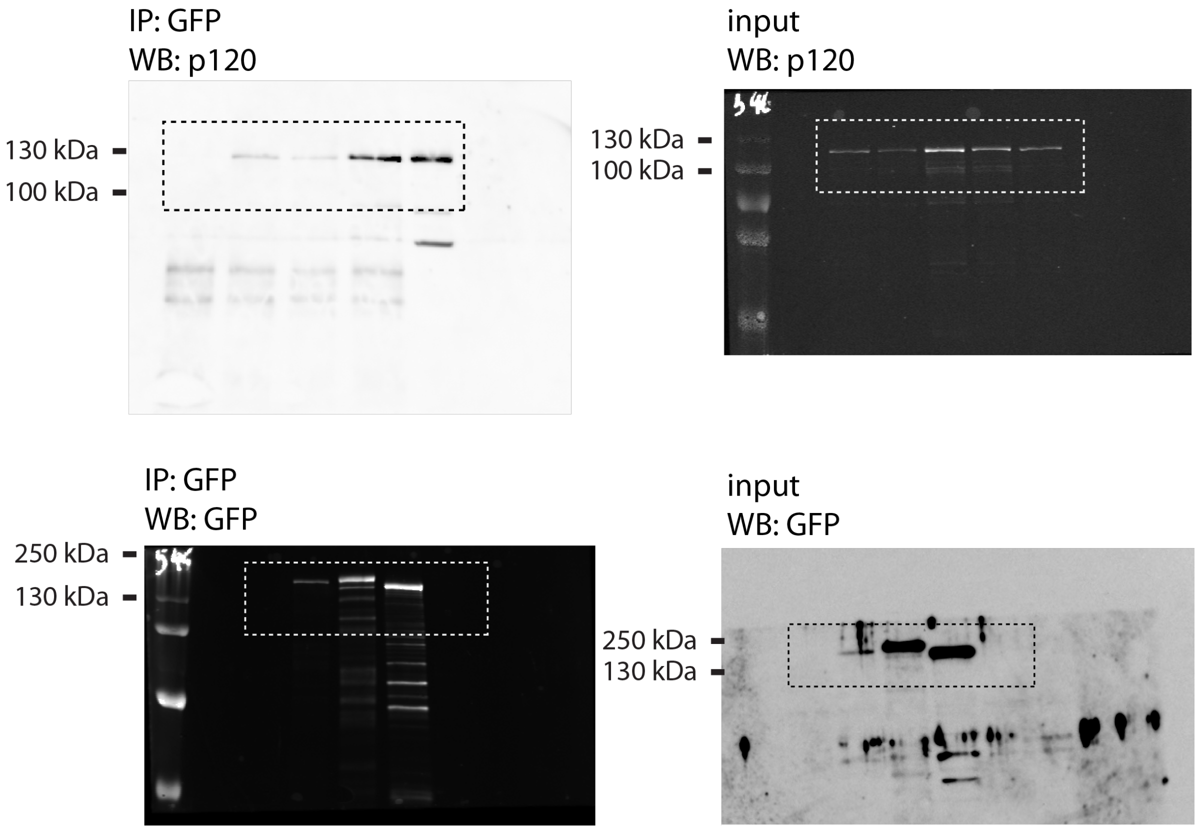

Supplementary Figure 7: Uncropped blots

This figure shows uncropped blots from the blots shown in the main and supplementary figures. Dotted boxes denote the area that is shown in the original figure.

Supplementary Table 1: Analysis of gross chromosomal content of Trp53$^{\Delta/\Delta}$-7, mILC-1 and PMC-het (*Trp53* $^{\Delta/\Delta}$;*Ctnnd1* $^{-/\Delta}$) and PMC-1 and -2 (*Trp53* $^{\Delta/\Delta}$;*Ctnnd1* $^{\Delta/\Delta}$).

**Cell line**	**Ploidy**
Trp53$^{\Delta/\Delta}$-7 (n=20)	2N (60%), 3N (5%), 4N (25%), 5N (10%)
mILC-1 (n=20)	2N (55%), 4N (20%), 5N (20%), 6N (5%)
PMC-het (n=20)	2N (0%), 3N (15%), 4N (80%), 8N (5%)
PMC-1 (n=20)	2N (10%), 4N (60%), 7N (20%), 8N (10%)
PMC-2 (n=20)	2N (5%), 3N (10%), 4N (60%), 5N (10%), 7N (5%), 8N (10%)
